# Early Th2 inflammation in the upper respiratory mucosa as a predictor of severe COVID-19 and modulation by early treatment with inhaled corticosteroids: a mechanistic analysis

**DOI:** 10.1016/S2213-2600(22)00002-9

**Published:** 2022-06

**Authors:** Jonathan R Baker, Mahdi Mahdi, Dan V Nicolau, Sanjay Ramakrishnan, Peter J Barnes, Jodie L Simpson, Steven P Cass, Richard E K Russell, Louise E Donnelly, Mona Bafadhel

**Affiliations:** a National Heart and Lung Institute, Imperial College London, London, UK; b National Institute for Health Research Oxford Biomedical Research Centre, Oxford, UK; c Nuffield Department of Medicine, University of Oxford, Oxford, UK; d School of Immunology and Microbial Sciences, Faculty of Life Sciences and Medicine, King's College London, London, UK; e UQ Centre for Clinical Research, The University of Queensland, Brisbane, QLD, Australia; f School of Mathematical Sciences, Queensland University of Technology, Brisbane, QLD Australia; g School of Medical and Health Sciences, Edith Cowan University, Perth, WA, Australia; h School of Medicine and Public Health, Priority Centre for Healthy Lungs, University of Newcastle, Callaghan, NSW, Australia

## Abstract

**Background:**

Community-based clinical trials of the inhaled corticosteroid budesonide in early COVID-19 have shown improved patient outcomes. We aimed to understand the inflammatory mechanism of budesonide in the treatment of early COVID-19.

**Methods:**

The STOIC trial was a randomised, open label, parallel group, phase 2 clinical intervention trial where patients were randomly assigned (1:1) to receive usual care (as needed antipyretics were only available treatment) or inhaled budesonide at a dose of 800 μg twice a day plus usual care. For this experimental analysis, we investigated the nasal mucosal inflammatory response in patients recruited to the STOIC trial and in a cohort of SARS-CoV-2-negative healthy controls, recruited from a long-term observational data collection study at the University of Oxford. In patients with SARS-CoV-2 who entered the STOIC study, nasal epithelial lining fluid was sampled at day of randomisation (day 0) and at day 14 following randomisation, blood samples were also collected at day 28 after randomisation. Nasal epithelial lining fluid and blood samples were collected from the SARS-CoV-2 negative control cohort. Inflammatory mediators in the nasal epithelial lining fluid and blood were assessed for a range of viral response proteins, and innate and adaptive response markers using Meso Scale Discovery enzyme linked immunoassay panels. These samples were used to investigate the evolution of inflammation in the early COVID-19 disease course and assess the effect of budesonide on inflammation.

**Findings:**

146 participants were recruited in the STOIC trial (n=73 in the usual care group; n=73 in the budesonide group). 140 nasal mucosal samples were available at day 0 (randomisation) and 122 samples at day 14. At day 28, whole blood was collected from 123 participants (62 in the budesonide group and 61 in the usual care group). 20 blood or nasal samples were collected from healthy controls. In early COVID-19 disease, there was an enhanced inflammatory airway response with the induction of an anti-viral and T-helper 1 and 2 (Th1/2) inflammatory response compared with healthy individuals. Individuals with COVID-19 who clinically deteriorated (ie, who met the primary outcome) showed an early blunted respiratory interferon response and pronounced and persistent Th2 inflammation, mediated by CC chemokine ligand (CCL)-24, compared with those with COVID-19 who did not clinically deteriorate. Over time, the natural course of COVID-19 showed persistently high respiratory interferon concentrations and elevated concentrations of the eosinophil chemokine, CCL-11, despite clinical symptom improvement. There was persistent systemic inflammation after 28 days following COVID-19, including elevated concentrations of interleukin (IL)-6, tumour necrosis factor-α, and CCL-11. Budesonide treatment modulated inflammation in the nose and blood and was shown to decrease IL-33 and increase CCL17. The STOIC trial was registered with ClinicalTrials.gov, NCT04416399.

**Interpretation:**

An initial blunted interferon response and heightened T-helper 2 inflammatory response in the respiratory tract following SARS-CoV-2 infection could be a biomarker for predicting the development of severe COVID-19 disease. The clinical benefit of inhaled budesonide in early COVID-19 is likely to be as a consequence of its inflammatory modulatory effect, suggesting efficacy by reducing epithelial damage and an improved T-cell response.

**Funding:**

Oxford National Institute of Health Research Biomedical Research Centre and AstraZeneca.

## Introduction

Understanding of the inflammation that occurs following SARS-CoV-2 infection has increased.^1^ Mainly, the immune response in patients with severe infection who require hospitalisation has been investigated.^2^ The early COVID-19 studies focused on inflammation caused by the systemic immune response^3^ whereas the inflammatory response in the airway of patients remains largely unknown, in part because of the risk of contagion through aerosolisation of the virus. The airway inflammatory response to common respiratory viruses in healthy individuals^4^ and in patients with chronic airways disease has been extensively studied.^5^, ^6^ Inhaled corticosteroids are often prescribed in patients with asthma and chronic obstructive pulmonary disease (COPD) to reduce the risk of exacerbations, which are usually mediated by respiratory viruses.^7^, ^8^, ^9^ During the ongoing COVID-19 pandemic, it has been reported that patients with asthma and COPD are less likely to be hospitalised with severe COVID-19 than people with comorbidities (eg, cardiovascular, diabetes, hypertension, and obesity).^10^, ^11^ The reasons for this observation are unclear but, in patients with asthma, the use of inhaled corticosteroids 2 weeks before hospitalisation for severe COVID-19 was associated with better clinical outcomes.^12^ Our recent STOIC study^13^ investigated inhaled budesonide, a corticosteroid, as a treatment for early SARS-CoV-2 infection with positive findings in improvement of self-reported symptom recovery, with fewer patients experiencing adverse outcomes and less symptom persistence. This finding was replicated in a large phase 3 efficacy trial (PRINCIPLE),^14^ but has yet to be adopted globally. The mechanism for how inhaled corticosteroids can improve early COVID-19 is currently unknown.

**Research in context**

**Evidence before this study**

Almost all patients with COVID-19 have an early prodromic illness and some develop severe COVID-19 illness requiring hospital care. There is a widespread view that the symptoms in early COVID-19 reflect a viral replication phase, whereas the severe hospitalised phase of the illness reflects an inflammatory phase of COVID-19. We searched PubMed with search terms “early COVID-19”, “SARS-CoV-2”, “respiratory inflammation”, “inhaled steroids”, “mechanism”, and “clinical trials”, from database inception to July 19, 2021, for studies published in English. Two community based randomised clinical trials (STOIC and PRINCIPLE) have shown that treating patients within 7 or 14 days of symptom onset with inhaled budesonide reduced the time to symptom resolution and reduced the risk of increase health-care resource utilisation. The early respiratory inflammatory manifestation following SARS-CoV-2 virus infection and mechanism of efficacy of inhaled budesonide is unknown.

**Added value of this study**

To our knowledge, the STOIC study is the only study to have sampled the nasal mucosa in early COVID-19, both at the early stages of the illness and following 2 weeks of study follow-up. Sampling was done routinely and prospectively on all trial participants, avoiding any sampling bias. Contrary to the prevailing view that inflammation occurs in the later, more severe stage of disease, there is a marked inflammation in the airway in early COVID-19. This effect was observed across viral response proteins, and T-helper 1 (Th1) lymphocyte and T-helper 2 (Th2) lymphocyte pathways. In patients with COVID-19 not treated with budesonide, there was persistently raised interferon and eosinophil chemokines. Interestingly, in patients with COVID-19 who met the primary endpoint (ie, clinical deterioration measured by urgent care visits), there was a muted early inflammatory response, except for raised eosinophil chemokines, followed by a severe second peak of inflammation. Budesonide treatment attenuated the eosinophil chemokine-driven inflammation, reduced the peak of the interferon and Th1 and Th2 lymphocyte-related inflammatory pathways, in addition to modulating inflammation in the respiratory tract and in the circulation. To our knowledge, this is the first study on the nasal mucosa and inflammatory markers in early disease and the first to investigate the mechanism of treatments used to treat COVID-19, which has determined potential biomarkers that can predict worsening COVID-19.

**Implication of the all the available evidence**

Many drugs were repurposed during the COVID-19 pandemic. Although some of these medications have been shown to be effective, there is little known about the mechanistic mode of action. This study provides further scientific validity to the STOIC trial, with prospectively and longitudinally collected airway samples showing that inhaled budesonide effectively altered the airway mucosal and the systemic inflammatory pattern. Inhaled corticosteroids are therefore efficacious in treating early COVID-19.

Here, we report the nasal mucosal inflammatory response in patients with early COVID-19 disease and examine the evolution of inflammation in the natural course of COVID-19 in patients from the STOIC study. We also identify how inflammation in the airway can predict illness severity and investigate the effect of inhaled budesonide on the respiratory mucosa in early COVID-19 disease. Finally, we show, by use of network analysis, how inhaled budesonide can resolve the exaggerated inflammatory response observed in early SARS-CoV-2 infection and aim to restore health.

## Methods

### Study design and participants

In this experimental analysis, we report on measures of inflammation using nasal mucosal lining fluid samples and serum samples collected during the STOIC trial. STOIC was a randomised, open-label, parallel group, phase 2 clinical intervention trial, which has been previously published.^13^ In brief, participants aged 18 years or older with early COVID-19 symptoms (defined as new onset of cough, fever, anosmia, or a combination of these symptoms for less than 7 days) were randomly assigned (1:1) to receive usual care (as needed antipyretics) or inhaled budesonide at a dose of 800 μg twice a day plus usual care. Participants were seen by nurses at home at randomisation (day 0) and day 14, when self-performed nasosorption was collected. At day 28, whole blood was collected. Full details of the study design can be found in the appendix (p 4). Participants provided written informed consent. The STOIC study was approved by the Fulham London Research Ethics Committee (20/HRA/2531) and the National Health Research Authority.

Healthy controls were adults aged 18 years or older without any known history of lung disease or COVID-19 symptoms (including negative COVID-19 antigen tests done on the day of nasal epithelial lining fluid and blood sampling), recruited from a long-term observational data collection study at the University of Oxford (ethics reference 18/SC/0361). Healthy controls were used as a comparator to evaluate inflammation in the healthy upper respiratory tract.

### Procedures

For this experimental analysis, nasal mucosal lining fluid was collected at days 0 and day 14 after randomisation with a nasosorption FX.I device (Hunt Developments UK, London, UK), consisting of a synthetic absorptive matrix strip against the inferior turbinate, for 1 min. Whole blood was collected at day 28 after randomisation and was allowed to clot for 60 min at room temperature. RNA was extracted using the QIAamp Viral RNA Mini Kit (Qiagen, MD, USA) following manufacturer's instructions, and testing for SARS-CoV-2 infection was done by quantitative real-time RT-PCR. Full details of sample collection and processing is in the appendix (p 1).

Immunoassays of mediators were selected to represent putative mechanistic inflammatory pathways associated with respiratory virus infections, and thus COVID-19,^15^, ^16^, ^17^ and were quantified using MSD (Meso Scale Diagnostics, Rockville, MD, USA). All values at or below the lower limit of detection (LLOD) or above the upper limit of detection (ULOD) were replaced by the LLOD or ULOD value as suggested by the assay parameters and are shown in the appendix (p 16). The mediator transforming growth factor beta-1 (TGFB1) was not done in the control samples because of assay unavailability.

### Outcomes

We investigated the nasal mucosal inflammatory response in patients with early COVID-19 disease from samples collected in the STOIC study at an early timepoint of disease onset (ie, within 7 days of symptoms) and compared it with the nasal mucosal inflammatory response in healthy controls. We also assessed the natural course of inflammatory response by evaluating resolution of inflammation in participants of the STOIC study comparing inflammation at an early timepoint and 14 days after study randomisation. Our analysis provides insight of mucosal inflammation following SARS-CoV-2 infection, which is predictive of worsening of COVID-19 illness. Finally, we studied the effect of treatment with inhaled budesonide on nasal mucosal inflammation compared with usual care and determined how budesonide resolves the exaggerated inflammatory response.

### Statistical analysis

To illustrate the temporal trends in inflammatory mediator concentrations in the usual care and budesonide study groups, we plotted smoothed splines of longitudinal mediator concentration data, using raw pg/mL data and the date from which symptoms started. This spline best-fit curve analysis assesses kinetic inflammatory changes over the disease course of early COVID-19 disease (represented in this study by the usual care study group) and with inhaled corticosteroids intervention (budesonide study group). Spline analysis considers the timing of the first symptom and gives an indication of peak of inflammation and resolution. The spline analysis was performed on all inflammatory mediators. Heatmaps were drawn to visualise data as a magnitude of change of all inflammatory mediators between healthy controls, early COVID-19 disease (day 0), and longitudinal (day 14) follow-up in usual care and budesonide treatment study groups. Inflammatory mediator data were standardised between mediators by calculating Z scores (using mean and SD from raw mediator values [pg/mL]).^18^

Volcano plots were drawn to illustrate in a scatterplot the magnitude of change (fold change, x-axis), against the statistical significance of that change (log_10_ of p value, y-axis). These were drawn for all mediators against the following conditions: comparison of inflammation in health and early COVID-19 (healthy controls against early COVID-19); the evolution of inflammation in the usual care study group (day 0 and day 14); the evolution of inflammation in the budesonide study group (day 0 and day 14); and comparison of day 0 inflammation in patients that deteriorated with COVID-19 (reaching primary endpoint) compared with those who did not deteriorate. Mann-Whitney or Wilcoxon signed-rank test was performed for unpaired or paired analysis for the magnitude of change. Serum samples were analysed with the Kruskall-Wallis test with post-hoc Dunn's multiple comparison test performed on all mediators.

To further elucidate the patterns of interactions between all inflammatory mediators in COVID-19 in the usual care and budesonide study groups, network analysis and visualisation software were developed in-house using MATLAB version 2021. We studied correlation matrices of mediator responses across the study groups, removing the noise eigenvalues and thus building up error-free mediator–mediator interaction networks. We then analysed these matrices to find modules of mediators that are interconnected with each other and relatively unconnected to other modules.

p values of less than 0·05 were considered statistically significant. All statistical analyses used GraphPad Prism version 9.0.0 and MATLAB version 2021. Full details of the network statistical analysis are presented in the appendix (p 2).

The STOIC trial was registered with ClinicalTrials.gov, NCT04416399.

### Role of the funding source

The funder of the study had no role in study design, data collection, data analysis, data interpretation, or writing of the report.

## Results

The STOIC study recruited 146 participants aged 18–79 years (mean 45 years [SD 13]) with a median duration of COVID-19 symptoms of 3 days (IQR 2–5). 140 nasal mucosal samples were available at day 0 (randomisation, visit 1) and 122 samples after 14 days (visit 3). At day 28 (visit 4), whole blood was collected from 123 participants (62 in the budesonide group, 61 in the usual care group). The healthy control cohort consisted of 20 samples from 20 healthy individuals. The demographics of the healthy volunteers and COVID-19 STOIC participants are presented in the appendix (p 15). Time to self-reported clinical recovery for all participants in the STOIC study was a mean of 9 days (SD 6).

**Figure 1:**
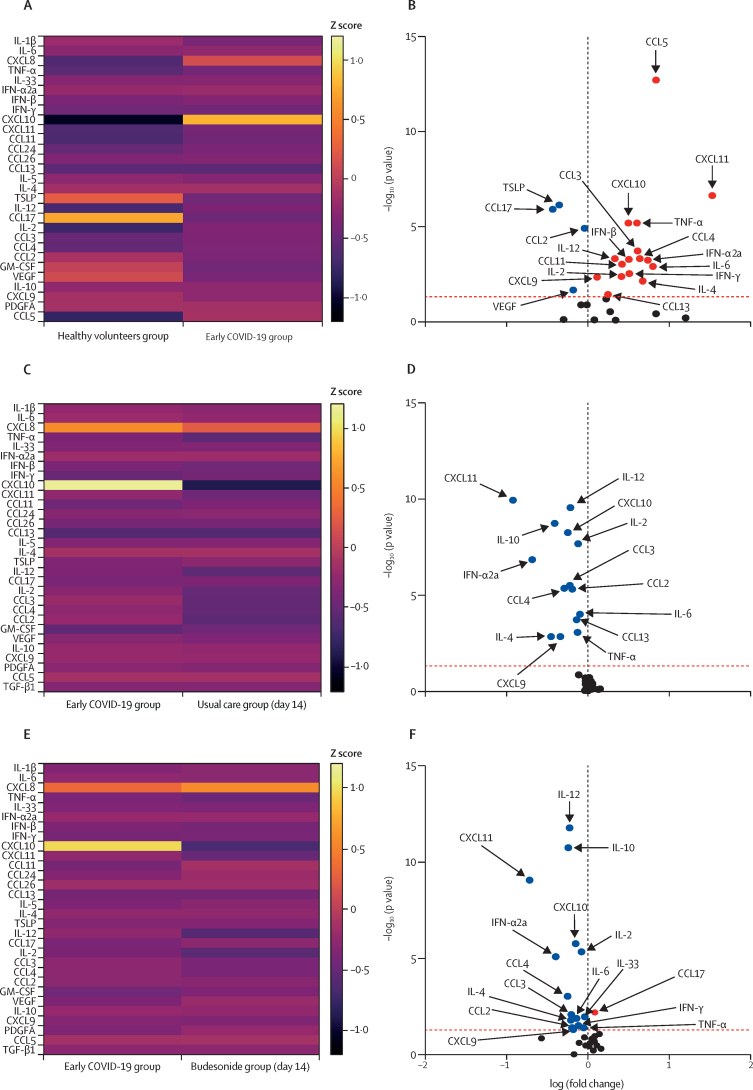
Immunological features of the nasal mucosa in patients with early COVID-19 over time in the STOIC study A) Heatmap and B) volcano plot of 26 nasal mediators from 20 healthy individuals and 140 patients with community-based early COVID-19. C) Heatmap and D) volcano plot of 30 nasal mediators from patients with community based early COVID-19 at day 0 after enrolment in the trial and day 14 in the usual care group (n=60). E) Heatmap and F) volcano plot of 30 nasal mediators from patients with community based COVID-19 at days 0 and 14 in the budesonide group (n=62). Horizontal dotted line on volcano plots depicts the cutoff for statistical significance; the vertical dotted line represents the cutoff point determining whether mediator concentrations were higher significantly (right, red) or lower (left, blue) for day 14 samples compared with either healthy controls (unpaired) or paired day 14 samples. Black dots represent changes in mediator concentration. Data were analysed using Mann-Whitney t-test or Wilcoxon matched pairs signed rank test. IL=interleukin. TNF=tumour necrosis factor. IFN=interferon. TSLP=thymic stromal lymphopoietin. CXCL=CXC chemokine ligand. CCL=CC chemokine ligand. GM-CSF=granulocyte-macrophage colony-stimulating factor. VEGF=vascular endothelial growth factor. PDGF=platelet-derived growth factor. TGF=transforming growth factor.

We examined the inflammatory profile of nasal mucosal fluid in healthy controls and early COVID-19 (figure 1A). We found that 16 mediators were increased in all patients infected with COVID-19 in both treatment groups compared with controls. These mediators were T-helper-type 1 (Th1) cytokines (interleukin [IL]-2, IL-12, tumour necrosis factor [TNF]-α, and IL-6,), T-helper-type 2 (Th2) cytokines (IL-4), interferon response proteins (interferon [IFN]-α2a, IFN-β, IFN-γ, CXC chemokine (CXC)L10, CXCL9, and CXCL11), and other chemokines (CC chemokine ligand (CCL)3, CCL4, CCL5, CCL11, and CCL13; figure 1B). An altered T-cell response with statistically significantly reductions in concentrations of thymic stromal lymphopoietin (TSLP) and CCL17 was also found in these patients; CCL2 concentrations were also decreased, related to impaired monocyte recruitment (figure 1B). Patients with COVID-19 also had lower concentration of vascular endothelial growth factor (VEGF; figure 1B). Some mediators were unaltered (IL-1β, IL-33, CCL24, CCL26, IL-5, granulocyte-macrophage colony-stimulating factor [GM-CSF], and IL-10; figure 1B), highlighting less activation during early COVID-19. CXCL8 was unchanged between health and early COVID-19.

We examined nasal mucosal inflammation during the course of disease (ie, in the usual care group), examining paired samples from the usual care group population at day 0 and day 14 (n=60; figure 1C). Over the course of 14 days, concentrations of CXCL9, CXCL10, CXCL11, IL-12, IL-10, IL-2, IFN-α2a, CCL2, CCL3, CCL4, IL-6, IL-4, CCL13, and TNF-α statistically significantly decreased. Of the 16 mediators elevated in early COVID-19 compared with healthy controls, 11 decreased over time (CCL3, CCL4, TNF-α, IL-6, CCL13, IL-4, IFN-α2a, CXCL10, CXCL11, IL-2, and IL-12). CCL11, IFN-β, and IFN-γ did not significantly change between day 0 and day 14 and remained elevated after 14 days in patients with COVID-19 in the usual care group compared with healthy volunteers. VEGF, TSLP, and CCL17 concentrations were unchanged over time in patients with COVID-19, remaining lower than those measured in healthy volunteers, but CCL2 and IL-10 concentrations significantly reduced further compared with day 14 (figure 1C and figure 1D). IL-1β, CXCL8, IL-33, CCL24, CCL26, IL-5, and GM-CSF did not show any changes between the two study visits over time.

To investigate the potential mechanisms of how budesonide reduces time to clinical recovery in patients with COVID-19, we examined the concentrations of 30 mediators in paired day 0 and day 14 samples in the inhaled budesonide group of the STOIC trial (n=62, figure 1E). Of the mediators examined, those that were different to the natural course of early COVID-19 disease (as described for the usual care group), IL-33 and IFN-γ were significantly reduced, CCL17 concentrations were significantly increased, and CCL13 was no longer significantly reduced between the two visits (figure 1F). The delta change between paired visits in the usual care study group and the budesonide study group showed that budesonide treatment significantly reduced IL-33 and IFN-γ with increased concentrations of CCL17, whereas CCL13 was no longer significantly reduced (additional data in appendix p 5). As all day 0 samples were obtained before randomisation to the study groups, we also compared both the usual care group and budesonide group day 14 samples with all day 0 samples to assess the return to health. We found that, following treatment with inhaled budesonide, CCL5 was significantly reduced, but not GM-CSF; concentrations of IL-2 and IL-4 were sustained (ie, did not decrease), despite decreasing in the usual care group (appendix pp 6–8). As the median time to clinical recovery was 1 day shorter in the budesonide study treatment group than in with the usual care study treatment group, these changes in the concentrations of these mediators might explain differences in the recovery and the part played in the disease course of COVID-19.

SARS-CoV-2 viral load and nasal mucosal mediator measurements were available in 122 paired samples at randomisation (day 0). We found that IFN-α2a, CXCL10, CXCL11, IL-12, CCL2, and IL-6 correlated with viral load (appendix p 17).

**Figure 2:**
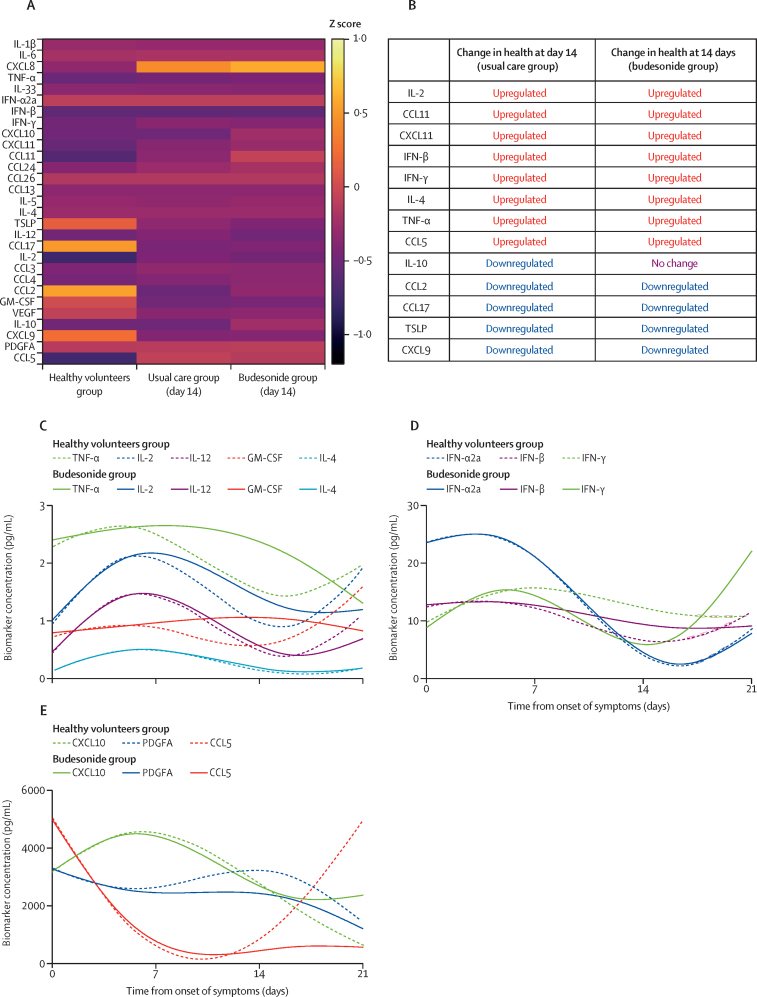
Temporal changes in mediator concentrations in the usual care and budesonide groups A) Heatmap of 26 nasal mediators from 20 healthy individuals compared with samples at day 14 after recruitment (day 14) in the usual care group (n=60) and the budesonide group (n=62). B) List of significantly altered mediators compared with healthy controls at day 14 in both usual care and budesonide groups. C–E) Longitudinal analysis of mediator profiles in the usual care and budesonide groups displayed as representative best fit curves by smoothed spline analysis. Additional data are presented in appendix (p 9). IL=interleukin. TNF=tumour necrosis factor. IFN=interferon. TSLP=thymic stromal lymphopoietin. CXCL=CXC chemokine ligand. CCL=CC chemokine ligand. GM-CSF=granulocyte-macrophage colony-stimulating factor. VEGF=vascular endothelial growth factor. PDGF=platelet-derived growth factor. TGF=transforming growth factor.

To assess whether the inflammation associated with SARS-CoV-2 infection declined over time to concentrations similar to healthy individuals, we compared our healthy cohort to the day 14 samples of the usual care and budesonide study groups (figure 2A). In both usual care and budesonide groups, compared with healthy controls, IL-2, CCL11, IL-4, TNF-α, IFN-β, IFN-γ, and CXCL11 remained significantly elevated over time, whereas CCL3, CCL4, IL-6, CCL13, IFN-α2a, CXCL11, and IL-12 reduced to comparable healthy concentrations (figure 2B). CCL2 and TSLP remained significantly lower in both study groups than in healthy volunteers. The concentration of CCL17 was increased in the budesonide study group over time and did not return to healthy concentrations. IL-10 significantly decreased over time in the usual care study group but not in the budesonide study group (figure 2B), indicating a sustained anti-inflammatory response in patients treated with inhaled budesonide.

In general, temporal trends in mediator concentrations in each group showed that peak inflammation occurred early in the disease course (figures 2C–E, appendix p 9). Additionally, IL-4, IL-12, IL-2, CXCL10, platelet derived growth factor subunit A (PDGFA), and IFN-α2a kinetic changes were similar between the natural course of early COVID-19 and inhaled budesonide. However, inhaled budesonide supressed kinetic inflammation and in particular a secondary increase in inflammation beyond day 15 (as seen with TNF-α, GM-CSF, and CCL5). IFN-γ increased following inhaled budesonide at day 15.

**Figure 3:**
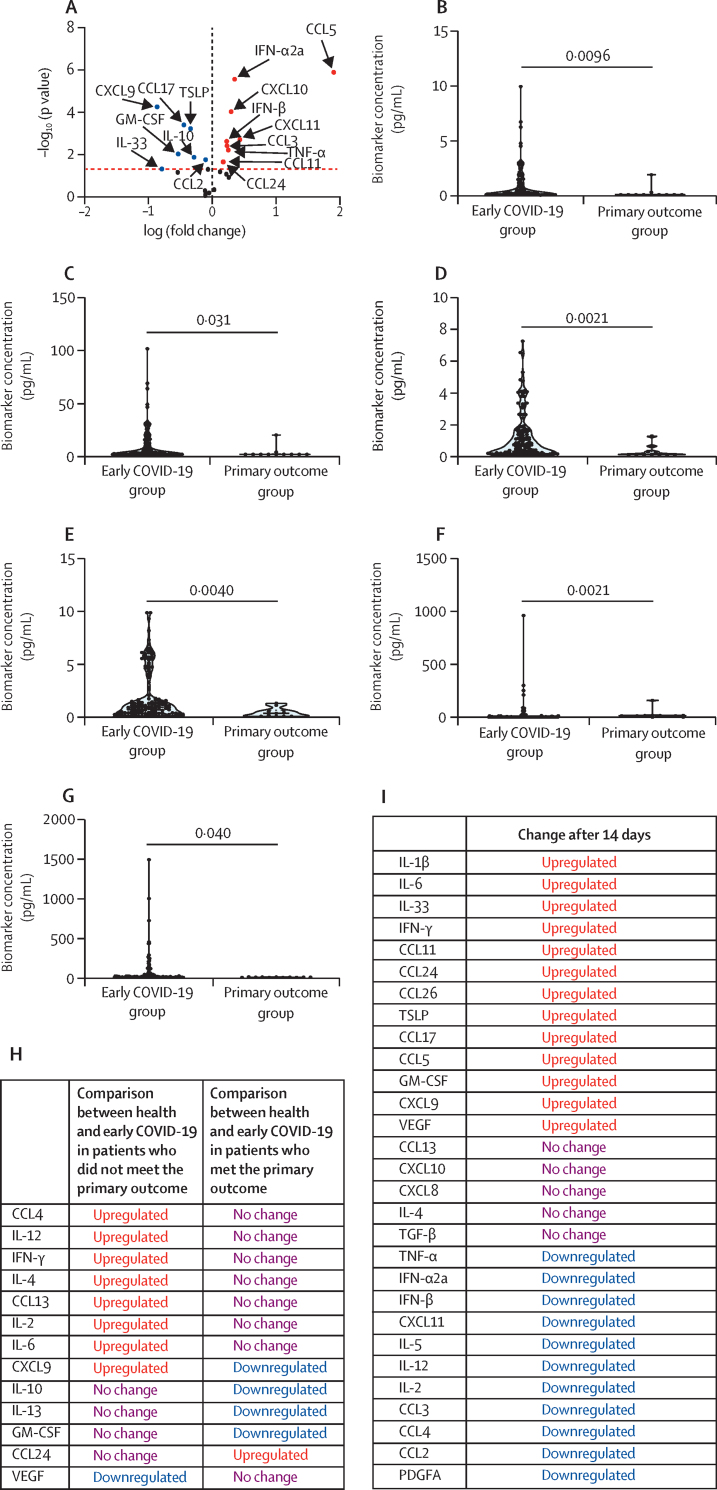
Alterations in nasal mucosal inflammation in patients with early COVID-19 who clinically deteriorate A) Volcano plot of 26 nasal mediators from 20 healthy individuals and 11 patients who met the primary outcome of the study. Red dots represent significantly increased mediator concentrations, blue dots sifnificantly decreases, and black dots no changes. B–G) Significantly altered mediators at day 0 from patients with early COVID-19 (n=129) and those who met the primary outcome (n=11). B) GM-CSF. C) IL-10. D) IL-12. E) IL-2. F) IFN-α2a. G) IL-33. H) Comparison table of significantly altered mediators from nasal samples comparing healthy volunteers (n=20) and patients with early COVID-19 (n=129) without deterioration and with COVID-19 with deterioration (n=11). I) Change in concentrations of 30 mediators from day 0 to day 14 of 1 patient who required critical care respiratory support (see appendix p 12 for heatmap). Data were analysed using Mann-Whitney t-test. IL=interleukin. TNF=tumour necrosis factor. IFN=interferon. TSLP=thymic stromal lymphopoietin. CXCL=CXC chemokine ligand. CCL=CC chemokine ligand. GM-CSF=granulocyte-macrophage colony-stimulating factor. VEGF=vascular endothelial growth factor. PDGF=platelet-derived growth factor. TGF=transforming growth factor.

11 participants met the primary outcome for deterioration in the STOIC clinical trial (defined as needing urgent care assessment, emergency care consultation, hospitalisation, or a combination of these outcomes), with 70% needing oxygen treatment. In comparison with healthy individuals, participants with early COVID-19 who met the primary outcome (ten in the usual care group and one in the budesonide group) had an increase of CCL3, CCL5, TNF-α, IFN-α2a, IFN-β, CXCL10, CXCL11, CCL11, and CCL24, but a reduction of CCL2, TSLP, CCL17, IL-10, GM-CSF, CXCL9, and IL-33 (figure 3A). However, there was an initial differential mediator response in participants who deteriorated compared with those who did not deteriorate following SARS-COV-2 infection. Firstly, there was no increase in CCL4, IL-12, IFN-γ, IL-4, CCL13, IL-2, and IL-6 in patients who deteriorated (figure 3H), showing an impaired inflammatory response. Secondly, there was a reduction in IL-10, IL-33, and GM-CSF in the primary outcome group. Finally, CCL24 concentrations were increased in participants who met the primary outcome, confirming that a high eosinophilic phenotype is associated with severe disease. Specifically, in participants who had clinical deterioration, the time course spline analysis showed that there was an aberrant immune response during the early part of SARS-CoV-2 infection (appendix pp 10–11), with significant downregulation of GM-CSF, IL-10, IL-12, IL-2, IFN-α2a, and IL-33 (figure 3B–G), suggesting a blunted early response to infection occurs in patients who then deteriorate. In one participant, who needed critical care support (usual care study group), longitudinal nasal sampling was available giving insight into course of the disease in a more severe patient. The interferon response proteins, CCL5, CXCL9, and CXCL10, remained elevated and CXCL11 decreased. Concentrations of the inflammatory markers IL-1β, IL-33, and IL-6 also increased over time (figure 3I, appendix p 12), illustrating persistent inflammation and epithelial damage.

**Figure 4:**
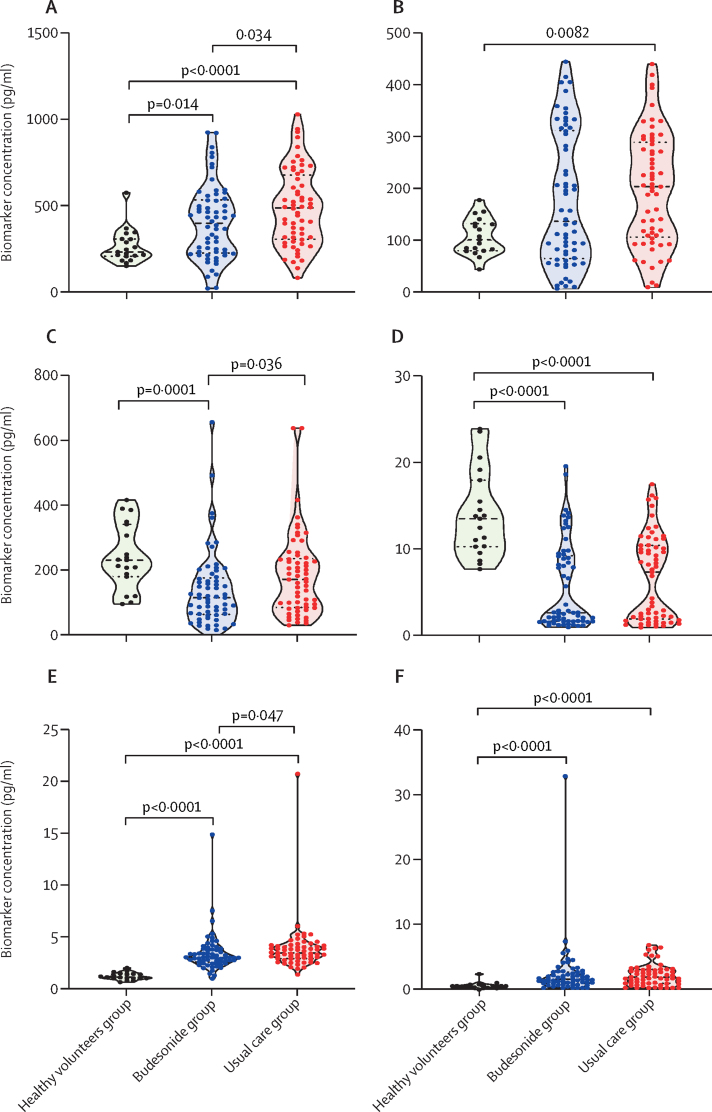
Persistence of systemic inflammation following 28–35 days of COVID-19 in the community Violin plots comparing some mediator concentrations in the serum of healthy individuals (n=20), those in the usual care group (n=61), and those in the budesonide group (n=62) of the study at 28–35 days following COVID-19 (see appendix p 13 for further results). (A) CCL11. (B) CCL13. (C) VEGF. (D) TSLP. (E) TNF-α. (F) IL-6. Data were analysed by Kruskal-Wallis with post-hoc Dunn's test. IL=interleukin. TNF=tumour necrosis factor. TSLP=thymic stromal lymphopoietin. CCL=CC chemokine ligand. VEGF=vascular endothelial growth factor.

Serum inflammatory mediators up to 35 days after initial infection remained elevated compared with healthy controls (figure 4A–F, appendix p 13). These mediators included higher concentrations of TNF-α, CXCL8, and the family of eosinophil chemotactic proteins (CCL11, CCL24, CCL26). This systemic inflammation was supressed to a greater degree by inhaled budesonide for CCL11, TNF-α, and IL-33.

**Figure 5:**
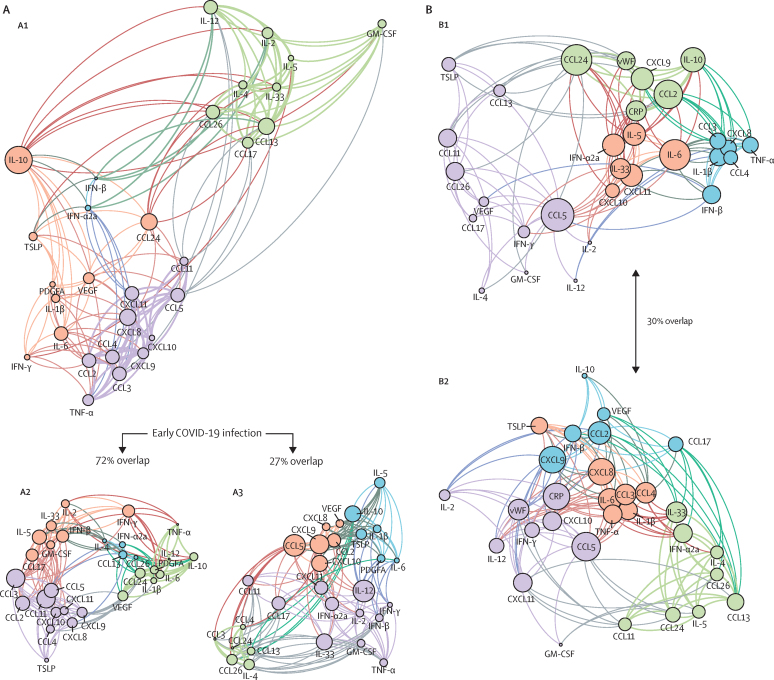
Network analysis of mediator correlation data (A) Modularity maximisation and community detection for eigenvalue-based noise-cleaned nasal mucosal mediator data. (A1) Networks at initial SARS-CoV-2 infection. (A2) Networks after 14 days of initial SARS-CoV-2 infection. (A3) Networks after treatment with inhaled budesonide. Node size indicates eigenvalue centrality and arcs indicate non-zero noise-cleaned correlation. There are four modules of roughly equal size in each network. A1 and A2 are similar with 72% overlap indicating persistent relationship of inflammation over time. A1 and A3 have 27% overlap (which would be expected by a chance reassignment of nodes to the four modules) indicating that treatment with inhaled budesonide changes the inflammatory networks entirely. (B) Modularity maximisation and community detection for eigenvalue-based noise-cleaned serum data in participants from 28 to 35 days following SARS-CoV-2 infection. (B1) Network analysis of serum from participants in the usual care group. (B2) Network analysis of serum from participants in the budesonide group. The two networks are entirely different in terms of membership with 30% overlap (equivalent to a random node reassignment to modules). The network analysis shown here suggests that budesonide treatment dramatically rearranged the mediator pathways in serum. CCL=CC chemokine ligand. CRP=C-reactive protein. CXCL=CXC chemokine ligand. GM-CSF=granulocyte-macrophage colony-stimulating factor. IFN=interferon. IL=interleukin. PDGF=platelet-derived growth factor. TGF=transforming growth factor. TNF=tumour necrosis factor. TSLP=thymic stromal lymphopoietin. VEGF=vascular endothelial growth factor. vWF=von Willebrand factor.

Network analysis of inflammatory mediators in the nasal mucosa and serum showed an anti-inflammatory response (IL-10 upregulation) and T2 inflammatory (CCL11 and CCL24 upregulation) response with co-existent inflammation (upregulation of IL-6 and CXCL8) in early COVID-19 (figure 5A-1). 2 weeks after initial infection, the natural course of COVID-19 shows persistent nasal mucosal inflammation with an exaggerated T2 inflammatory response (CCL11 and IL-5) and a sustained pro-inflammatory response (figure 5A-2). In the usual care study group, network analysis showed a 72% overlap between the inflammatory response during the first 7 days of COVID-19 symptom onset and at day 14 after randomisation, indicating persistent inflammation of similar immune pathways. Following inhaled budesonide treatment, the nasal mucosal response is different with an increased anti-inflammatory response (IL-10) and alarmins (IL-33 and TSLP), but a reduced T2 inflammatory response highlighting normalisation of the anti-viral immune response and the T2 hyper-inflammatory response. The overlap between the inflammatory response at an early timepoint in COVID-19 and at the later timepoint was only 27% in the budesonide study group (figure 5A-3). Network analysis of serum mediators performed between 28 and 35 days after initial infection in the usual care study group shows persistent IFN-α2a, CCL11, and CCL2 (figure 5B-1). There was only a 30% overlap with the serum of participants that had been treated with inhaled budesonide (figure 5B-2) again indicating immunomodulation.

## Discussion

In this study, we examined the inflammatory effect of SARS-CoV-2 on the upper airway at a very early timepoint during the course of COVID-19 disease^13^ and followed patients over the evolutionary course of SARS-CoV-2 infection. Our findings show that there is an early activated and enhanced immune response in early COVID-19 in the upper airway. We have also shown that we might be able to predict which patients will clinically deteriorate, by noting that they have a blunted interferon and an exagerated CCL24 airway inflammatory response. This finding has value in the early identification, monitoring, and therapeutics of this population by using biomarkers from the nose to detect the patients who might get worse. We have also found that there is persistent systemic inflammation in COVID-19, which can be measured up to 35 days after the initial infection and past the median time of clinical recovery. Finally, network analysis has shown that patterns of inflammation and inflammatory pathways in the airway and circulation are modified significantly following treatment with inhaled budesonide. These findings suggest that inhaled budesonide modulates the inflammatory pathways in the upper respiratory tract and circulation following COVID-19 infection (appendix p 14).

Due to the nature of the STOIC study^13^ design, we were able to examine the kinetics of inflammation between patients with early COVID-19 and in patients who received inhaled budesonide as a treatment intervention. We found that, in early COVID-19, there is upregulation of inflammation, which does not return to levels observed in healthy patients over time. Furthermore, mediators such as VEGF were found to be lower in the respiratory tract in early disease as previously found in severe COVID-19.^19^ Examination of the mediator temporal relationship showed that there was often a biphasic peak of inflammatory mediators, which is staggered and persists over time. Importantly, however, we found that in patients given inhaled budesonide, the inflammatory peak was attenuated for TNF-α, GM-CSF, and CCL5, and promoted for IFN-γ. IFN-γ is an important promoter of anti-viral immunity^20^ playing a role in the inhibition of viral replication in cells,^21^ suggesting that this is another mechanism for the efficacy of inhaled corticosteroids. Following inhaled budesonide treatment, the nasal mucosal response is different from that of the usual care group with an anti-inflammatory response (IL-10), an increase in alarmins (IL-33 and TSLP), and a reduction in the Th2 inflammatory response, which suggests the promotion of a normal anti-viral response and suppression of the exaggerated Th2 inflammatory response.

Our study suggests, unlike previous assumptions,^22^ that there is a vigorous and early immune response in the upper airway in patients who develop COVID-19. This response, in comparison with healthy controls, is an early combined type I and type II interferon response, pro-inflammatory response, and anti-inflammatory response. Interestingly, early in COVID-19, there is also a Th2 immune response, mediated mainly by CCL11 and IL-4. This early COVID-19 innate cytokine immune response leads to recruitment of eosinophils, natural killer cells, and macrophages, and is an immune response similar to that observed with other respiratory viruses.^23^

We have found that, in patients in whom there was a clinical deterioration following SARS-CoV-2 infection, the initial inflammatory response in the airway is blunted with reduced concentrations of IL-2, IL-33, IL-12, and IFN-α2a, in comparison with patients with COVID-19 without deterioration. A blunted interferon response, as a feature of severe COVID-19, has previously been observed in the systemic circulation^24^ and from in-vitro cell line transcription expression.^25^ As our study focused in early COVID-19, these findings suggest that an early impaired interferon response to COVID-19 could indicate a poor prognosis, potentially offering a strategy for early treatment for this at risk patient population. The identification of patients early and at high risk of deterioration could markedly improve clinical care for patients. Our findings might also suggest why treatment trials enhancing or attenuating specific inflammatory mediators might have larger treatment effects if given early, with an even greater likelihood of success if targeted to patients with a biological high risk. Interestingly, CCL24 was the only mediator that increased in patients who deteriorated following SARS-CoV-2 infection. The CCL subfamily of eosinophil chemotactic proteins (CCL11, CCL24, and CCL26), along with IL-5, GM-CSF, and CCL5 are responsible for lung recruitment and activation of eosinophils.^26^ Recruitment of eosinophils to the lung is the most probable explanation for the reduced circulatory number of eosinophils observed as part of the clinical picture of severe COVID-19.^27^ Furthermore, inhaled corticosteroids are recognised to be therapeutically effective in patients with an eosinophilic phenotype of airways disease.^28^ The elevation of CCL24 in patients that subsequently deteriorated from our study validates that the high eosinophilic phenotype is associated with severe disease, which is in line with previously published findings.^15^

Our study is also the first to show that there is ongoing inflammation in the circulation, over 4 weeks after SARS-CoV-2 infection, with elevated concentrations of IL-2, IL-6, CXCL8, CCL13, TNF-α, CCL11, CCL24, and CCL26. Persistent symptoms following SARS-CoV-2 infection have been identified as an emerging problem (long COVID)^29^ and our study indicates that this issue might be underlined by persistent inflammation. To note, the airway and peripheral blood immune profile in severe COVID-19 is different from early infection.^30^ It is, therefore, plausible that the identification of inflammatory mediators in the systemic circulation is delayed compared with assessing samples proximal to the site of infection. The STOIC study showed that persistence of symptoms was significantly lower in patients given inhaled budesonide, suggesting that early inhaled corticosteroid treatment can improve symptoms of COVID-19 and might help to prevent the effects of long COVID. We found that TNF-α and CCL11 were significantly lower in the systemic circulation in patients who had been treated with inhaled budesonide compared with usual care, suggesting that these mediators could be key for future clinical targets, particularly against long COVID. Interestingly, patients with long COVID commonly report symptoms of breathlessness,^31^ and asthma has been found to be the only pre-existing comorbidity with an independent association of long COVID.^31^ Whether there is a pre-existing host or exaggeration of the Th2 phenotype that leads to long COVID warrants further investigation.

Our study provides a unique mechanistic insight into the host respiratory immune and inflammatory response to early COVID-19 and following a therapeutic intervention that has been shown to have clinical benefit in treating these patients.^13^, ^14^ Notably, studies thus far have examined only a small number of airway samples from severe and hospitalised patients, often late in the disease course^32^, ^33^ or measured the immune response in the circulation.^34^ Moreover, these studies have focussed on transcriptional analysis, namely gene expression, which might not fully capture the protein environment. In contrast, our study is the first to examine protein concentration in the upper respiratory tract of patients with early COVID-19, thus providing a mechanistic insight into the host respiratory immune and inflammatory response to early COVID-19. The number of participants studied was based on the STOIC sample size, calculated for assessment of a clinical treatment endpoint. Although we did not conduct a formal sample size for an immunological endpoint, we can confirm that this analysis was suitably powered and that for any one single mediator the sample size we have is on average twice the number needed for an α of 0·05 and a β of 0·20 (80% power).

Our study has some limitations. Firstly, we did not obtain daily samples, nor did we obtain samples from the lower respiratory tract. However, to our knowledge, this is the first study to evaluate the respiratory tract in early COVID-19 and it is well recognised that the greatest abundance of angiotensin-converting enzyme 2 receptors is in the nasal mucosa^35^ and thus our findings are relevant to the primary site of infection. Second, our analysis used raw mediator concentration data, and not log-transformed mediator data, which can overestimate the SD. However, this analysis was performed to avoid overinterpretation. Furthermore, we could not determine any changes in CXCL8 between health and early COVID-19 as would be expected, but this finding is likely to reflect assay sensitivity because CXCL8 was repeatedly over the limit of detection, occurring in over one third of healthy control samples and one half of COVID-19 samples. Third, the STOIC study was conducted in a community setting in Oxfordshire (UK) and we recognise that our population were mainly white, despite our attempts to recruit ethnic minorities via advertisements and social media in local minority communities, such as places of worship. We believe, however, these inflammatory findings are likely to be independent of ethnicity,^36^ but acknowledge further study is warranted. Fourth, our study has focused on inflammation in early COVID-19 and, therefore, does not allow the evaluation of severe symptoms and inflammation. Fifth, only 11 patients met the primary outcome for the STOIC trial, thus replication of CCL24 as an early biomarker for deterioration is warranted. Finally, we note that our healthy control sample size was small, in comparison to the STOIC sample size, but our evaluation here was primarily as a comparator. Recruitment of a bigger sample size of controls was limited due to assay availability and due to the third national lockdown in the UK. 21 (15%) of STOIC participants had a history of past or current (but untreated) asthma, whereas none of the healthy controls by definition had a respiratory comorbidity. Although, the absence of respiratory comorbidities in the healthy controls might be noted as a limitation, our findings do show that the Th2 response to SARS-CoV-2 is similar across both individuals with or without respiratory disease.

To conclude, the initial observation of a significant under-representation of patients with obstructive lung disease (asthma and COPD) with severe COVID-19 and the knowledge that treatment with inhaled corticosteroids is routinely used to prevent viral exacerbations was the founding hypothesis for the STOIC trial. We have observed that the clinical benefit of inhaled budesonide as treatment in early COVID-19 is a consequence of its inflammatory modulatory effect. Moreover, our study supports a potential airway biomarker to detect clinical deterioration of COVID-19 and possible therapeutic targets for long COVID.

## Data sharing

All data can be shared upon written request to the corresponding author. Matlab code will also be made available upon request.

**This online publication has been corrected. The corrected version first appeared at thelancet.com/respiratory on May 31, 2022**

## Declaration of interests

SR reports grants and non-financial support from Oxford Respiratory National Institute for Health Research (NIHR) Biomedical Research Centre (BRC), during the conduct of the study; and non-financial support from AstraZeneca and personal fees from Australian Government Research Training Program, outside of the submitted work. LED reports grants from AstraZeneca and Boehringer-Ingelheim, outside of the submitted work. PJB reports grants and personal fees from AstraZeneca and Boehringer Ingelheim, and personal fees from Teva and Covis, during the conduct of the study. REKR reports grants from AstraZeneca, and personal fees from Boehringer Ingelheim, Chiesi UK, and GlaxoSmithKline, during the conduct of the study. MB reports grants from AstraZeneca; personal fees from AstraZeneca, Chiesi, and GlaxoSmithKline; and is a scientific advisor for Albus Health and ProAxsis, outside of the submitted work. JRB, SPC, MM, DVN, and JSL declare no competing interests.
