# Supplementary material for: Early Th2 inflammation in the upper respiratory mucosa as a predictor of severe COVID-19 and modulation by early treatment with inhaled corticosteroids: a mechanistic analysis
Source: Lancet Respir Med. 2022 Jun;10(6):545–56. doi: 10.1016/S2213-2600(22)00002-9 (PMC8989397; doi:10.1016/S2213-2600(22)00002-9)
Supplement: Supplementary appendix [file mmc1.pdf]

THE LANCET
Respiratory Medicine

Supplementary appendix

This appendix formed part of the original submission and has been peer reviewed. We post it as supplied by the authors.

Supplement to: Baker JR, Mahdi M, Nicolau DV Jr, et al. Early Th2 inflammation in the upper respiratory mucosa as a predictor of severe COVID-19 and modulation by early treatment with inhaled corticosteroids: a mechanistic analysis. *Lancet Respir Med* 2022; published online April 7. https://doi.org/10.1016/S2213-2600(22)00002-9.

Supplementary Material

Supplementary methods

Nasal mucosal sampling

Nasal mucosal sampling was self-performed by all participants. Briefly, samples of the nasal mucosal lining fluid were collected by placing a Nasosorption™ FX·I device (Hunt Developments UK Ltd) consisting of a synthetic absorptive matrix strip against the inferior turbinate for a duration of 1 minute. The sample was then eluted in 500 µL of PBS, 1% bovine serum albumin (BSA) (w/v), 1% Triton X-100 (v/v), and 0.05% sodium azide (w/v) (Sigma-Aldrich, UK) and stored at -80°C.

Isolation of serum

Whole blood was collected into an SST tube (Fisher Scientific, Leicestershire, UK) and allowed to clot for 60 min at room temperature. The serum sample was then centrifuged at 3000 x *g* for 10 min at room temperature. Serum samples were then aliquoted and stored at -80°C.

Virus extraction and quantification

RNA was extracted using the QIAamp Viral RNA Mini Kit following manufacturer's instructions. Virologic testing for SARS-CoV-2 infection was performed by quantitative real-time RT-PCR (RT-qPCR). Briefly, 420 µl of sample was extracted and eluted into 40 µl Buffer AVE. 10 µl of eluted RNA was assayed using the Taqman fast virus 1-step master mix (ThermoFischer Scientific, Loughborough, UK), utilising oligonucleotide primers (600 nM forward and 800 nM reverse per reaction) and fluorescent conjugated probes (two probes 100 nM each) (Eurofins Genomics, Wolverhampton, UK) for the detection of the viral RNase P gene (RdRP) gene region of SARS-CoV-19. Quantification of virus RNA copies were generated using a standard curve using nCoV-WHO-Control plasmid of known concentration (Eurofins Genomics, Wolverhampton, UK).

Immunoassays

Immunoassays of mediators were selected to represent putative mechanistic inflammatory pathways associated with respiratory virus infections and thus COVID-19[1-3]. Granulocyte macrophage-colony stimulating factor (GM-CSF), vascular endothelial growth factor (VEGF), tumour necrosis factor (TNF)-α, interferon (IFN)-α2a, IFN-β, IFN-γ, IL-33, thymic stromal lymphopoietin (TSLP), and interleukin (IL-)--1β, IL-2, IL-4, IL-5, IL-6, CXCL8, IL-10, IL-12, CXCL10, CXCL11, CCL2, CCL3, CCL4, CCL13, CCL17, CCL11, CCL24 and CCL26 were quantified using MSD (Meso Scale Diagnostics, Rockville, Maryland, USA) 10 spot U-Plex plates. Transforming growth factor (TGF)-β1, platelet derived growth factor (PDGF)-A, CXCL9, CCL5, von-Willebrand factor (vWF) and high-sensitivity C reactive protein (hsCRP) were measured by individual V-Plex plates, on a SQ120 Quickplex instrument (Meso Scale Diagnostics, Rockville, Maryland, USA). All values at or below the lower limit of detection (LLOD) or upper limit of detection (ULOD) were replaced by the LLOD or ULOD value as suggested by the assay parameters. Nasal mucosal mediators that were consistently around the ULOD were CXCL10 (6000 pg/ml) and CXCL8 (2100 pg/ml) whilst IFN-α2a (4 pg/ml), CCL26 (7.3 pg/ml), IL-4 (0.08 pg/ml) and TGF-β1 (9.1 pg/ml) were around the LLOD.

Network analysis of inflammatory mediators

To further elucidate the patterns of interactions between inflammatory mediators in COVID-19 both with and without inhaled budesonide treatment, we performed a mathematical analysis of the networks of mediators in various subsets of our data.

First, we computed correlation coefficients between mediator values in the nasal data across patients. For each of *M* mediators, we have values for *n* patients in either the BUD or UC arms. Regarding each patient index as an independent variable and each mediator value as a dependent variable, we can compute mediator-mediator expression correlation values, yielding an *m* x *m* matrix, with 1s on the diagonal.

This matrix represents a network with *m* mediator nodes and whose entries correspond to the strength of mediator co-expression. In principle this network can be analysed as-is. However, it is known that correlations between data series containing measurement error contain as a result a large component of 'noise'. In particular, the eigenvalues of a rectangular matrix whose entries are random follow the Marchenko-Pastur distribution. Therefore, those eigenvalues (and their associated eigenvectors) for an *m* x *m* correlation matrix falling within this distribution correspond to noise in the data and should be removed from the matrix. Specifically, the eigenvalues falling between the bounds

$$\lambda_{\pm} = \left(1 \pm \sqrt{\frac{m}{n}}\right)^2$$

correspond to noise. In the types of correlation matrices analysed here (where *n* is not much larger than *m*), most eigenvalues fall between these bounds (i.e., most of a correlation matrix is actually noise), confirming that analysing the original correlation networks directly would yield highly unreliable findings. By setting the noise eigenvalues to zero and reconstituting the matrix from the remaining non-zero (signal-associated) eigenvalues and their eigenvectors, we get an "eigen-cleaned" matrix, which can be used to perform network analysis on the co-expression of mediators (code available on request). We performed this procedure for the following data sets:

- Early COVID-19 infection at study enrolment (visit 1, baseline) for nasal mucosal inflammatory data
- BUD and UC arm analysis at visit 3 (14 days after randomisation) for nasal mucosal inflammatory data
- Serum mediator data for participants 28-35days after COVID-19 infection in the BUD and UC study arms respectively

This procedure results in 6 mediator-mediator co-expression networks. We split the analysis into the nasal and serum components. In the former case, we can look at how the visit 3 UC and BUD networks differed from the early COVID-19 baseline network, as well as from each other. In the latter case, we can compare the UC and BUD visit 3 networks with each other as well as with the healthy control network. We used modularity maximisation (code available on request) to detect communities (modules) within each of these networks. We chose the well-known Louvain algorithm for this analysis[4] due to its robustness. Remarkably, in each of these networks, the algorithm detects four sub-modules that are highly internally connected and relatively unconnected from the other modules, of approximately equal size (4-8 mediators) confirming that there is substantial structure in mediator-

mediator co-expression. However, the mediator membership assignment to the modules in each case varies substantially and, in particular, is altered by treatment with budesonide versus usual care.

The study sample size is as previously described[5]. The study had a statistical power of >99% to show a budesonide treatment effect.

Supplementary Figures

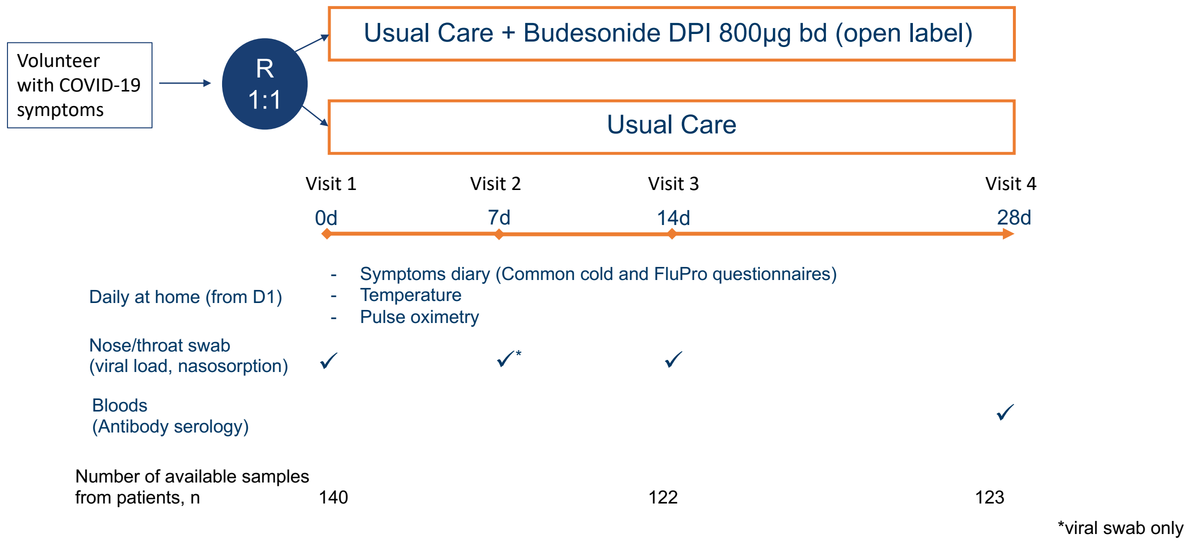

Figure S1. Study design schematic for the STOIC study. DPI Dry powdered inhaler; bd (twice per day); d days. Patient samples available at Visit 1 n=140; Visit 3 n=122 (4 samples destroyed in transport; 10 samples not collected as primary endpoint reached; 4 samples not available due to participant withdrawal); Visit 4 n=123 (4 samples not collected due to participant withdrawal; 11 samples not collected as primary endpoint reached; 2 samples not available due to insufficient sample)

Figure S2. Delta changes between Usual care and Budesonide arm. Significant Delta changes in protein levels between visit 1 and visit 3 between usual care arm and budesonide treated arm. Delta changes in A) IL-33 and b) CCL17 comparing the visit 3 samples from usual care (n=60) and budesonide (n=62). Data were analysed using Mann-Whitney t-test. *P<0.05

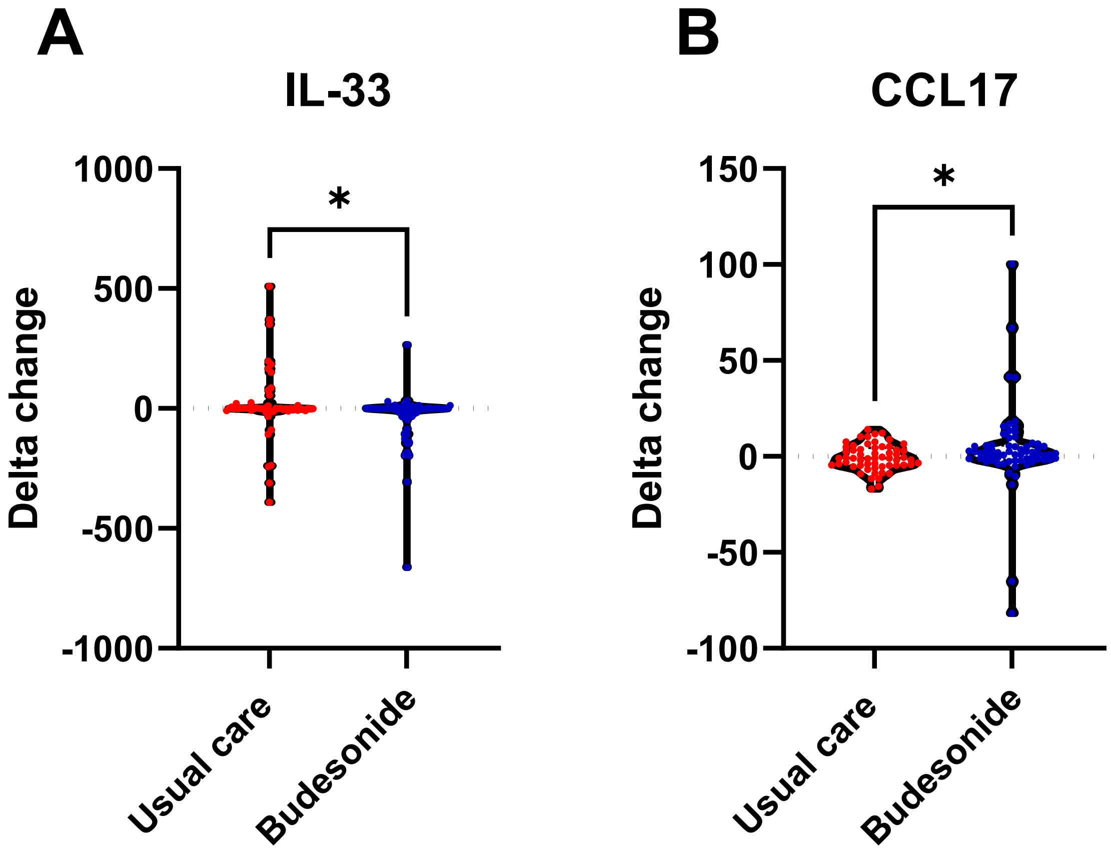

Figure S3. Difference in mediator levels between usual care and budesonide treatment compared to all early COVID-19 patients. A) A heatmap depicting changes in nasal mediators comparing levels in all visit 1 samples (n=140) and visit 3 in usual care arm (n=60) and budesonide arm (n=62). B) Volcano plot comparing all visit 1 samples to usual care visit 3. C) Volcano plot comparing all visit 1 samples to budesonide visit 3. ●= upregulated. ●= downregulated. Data were analysed using Mann-Whitney t-test.

Figure S3A

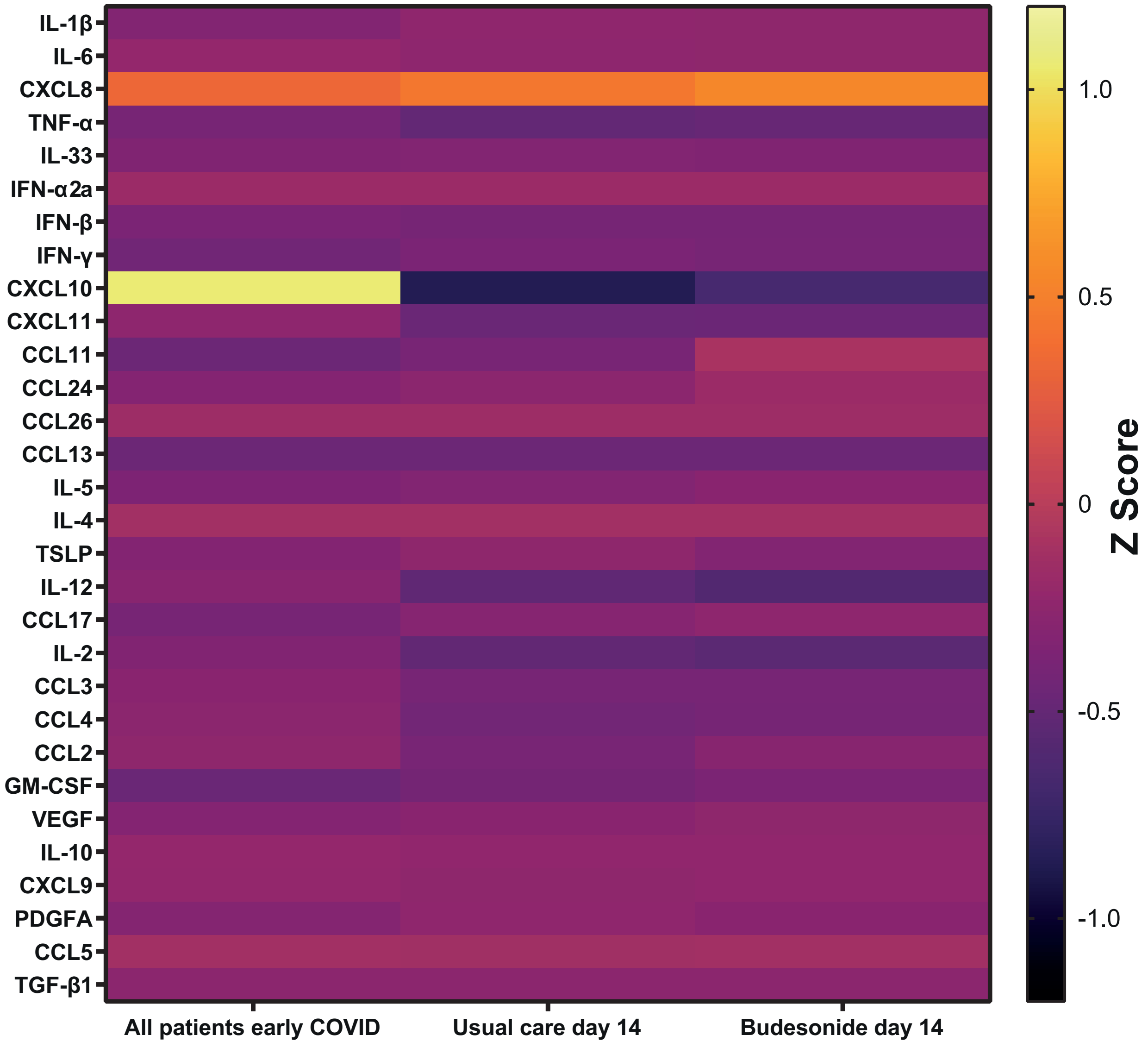

Figure S3B

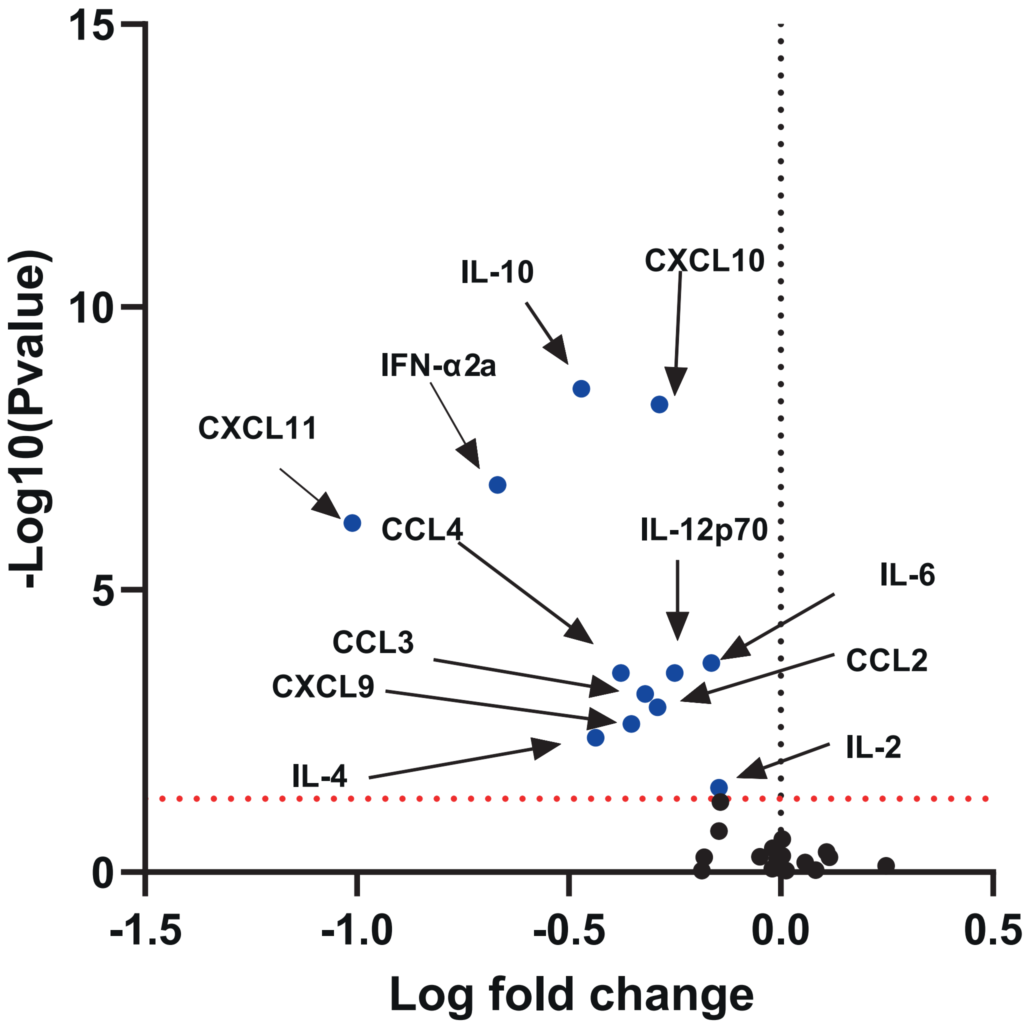

-Log10(Pvalue)
15
10
5
0
-1.5
-1.0
-0.5
0.0
0.5
Log fold change
IL-10
CXCL10
IFN-α2a
CXCL11
CCL4
IL-12p70
IL-6
CCL3
CCL2
CXCL9
IL-4
IL-2

Figure S3C

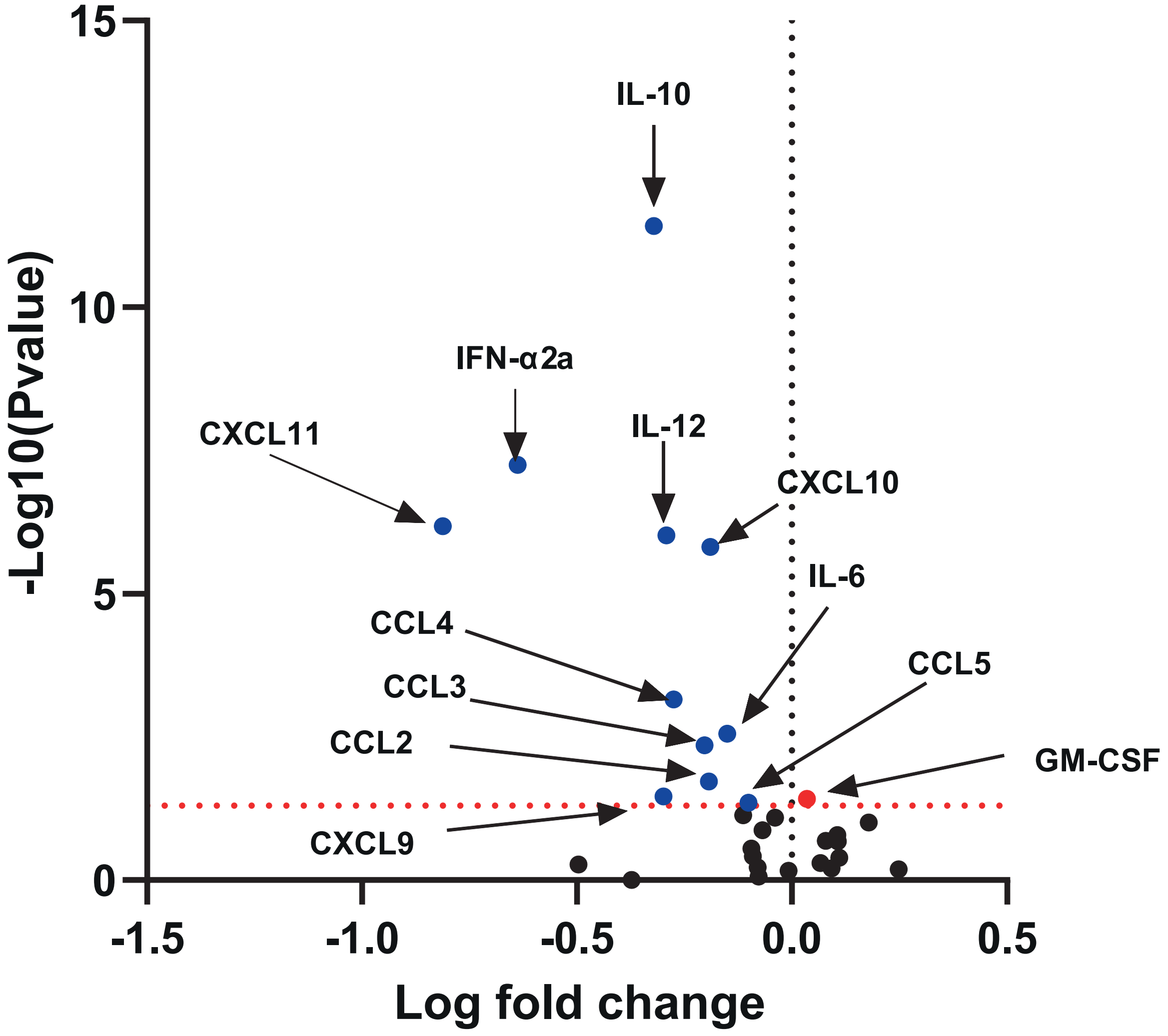

-Log10(Pvalue)
15
10
5
0
-1.5
-1.0
-0.5
0.0
0.5
Log fold change
IL-10
IFN-α2a
CXCL11
IL-12
CXCL10
IL-6
CCL4
CCL3
CCL5
CCL2
GM-CSF
CXCL9

Figure S4. Spline analysis of nasal mucosal mediator levels from first symptom onset. Longitudinal analysis of mediator profiles in the A) usual care (n=60) and B) budesonide arm (n=62) displayed as representative best fit curves by smoothed spline analysis.

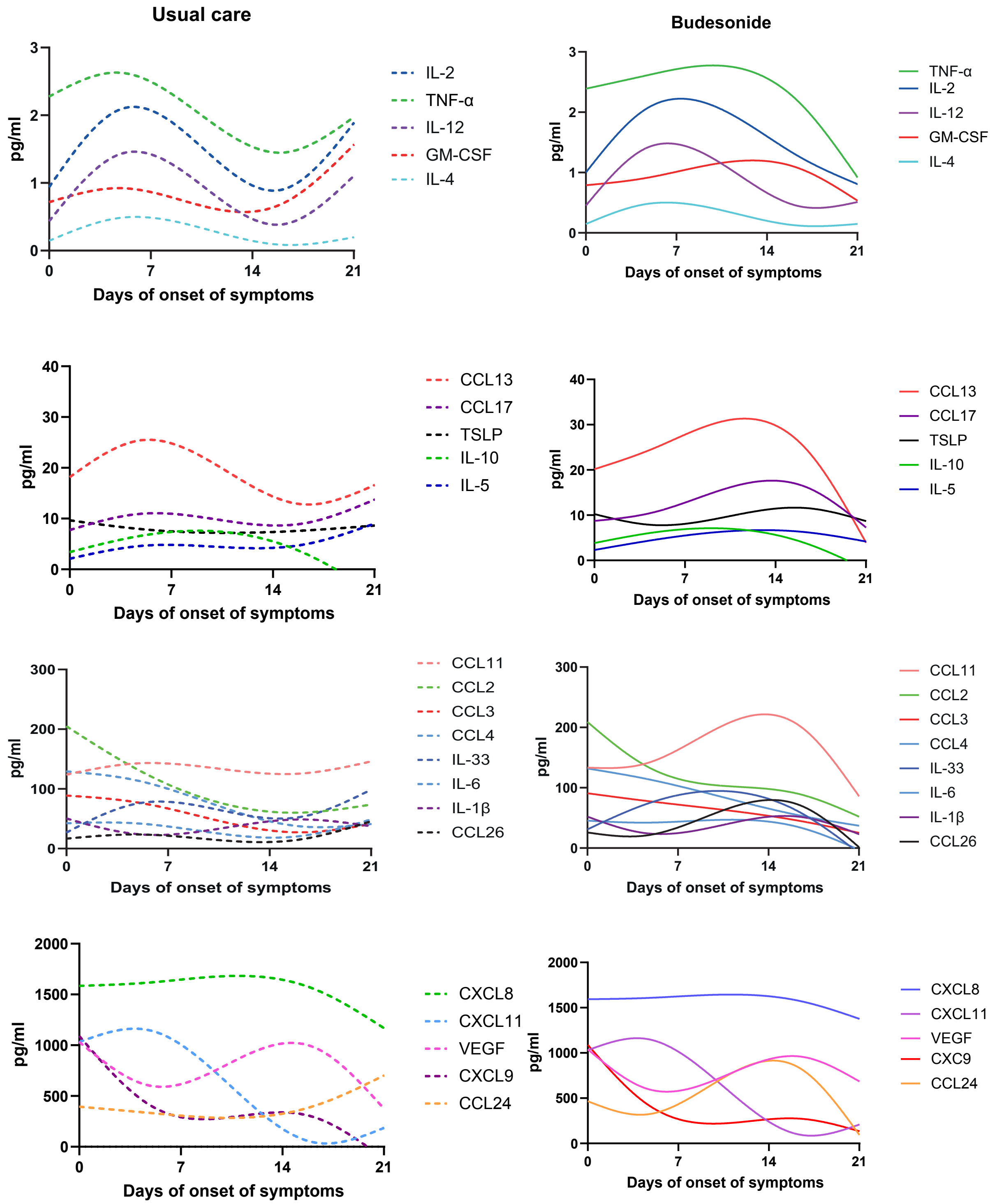

Figure S5. Spline analysis of nasal mucosal mediator levels from first symptom onset in participants who reached the primary outcome (n=11).

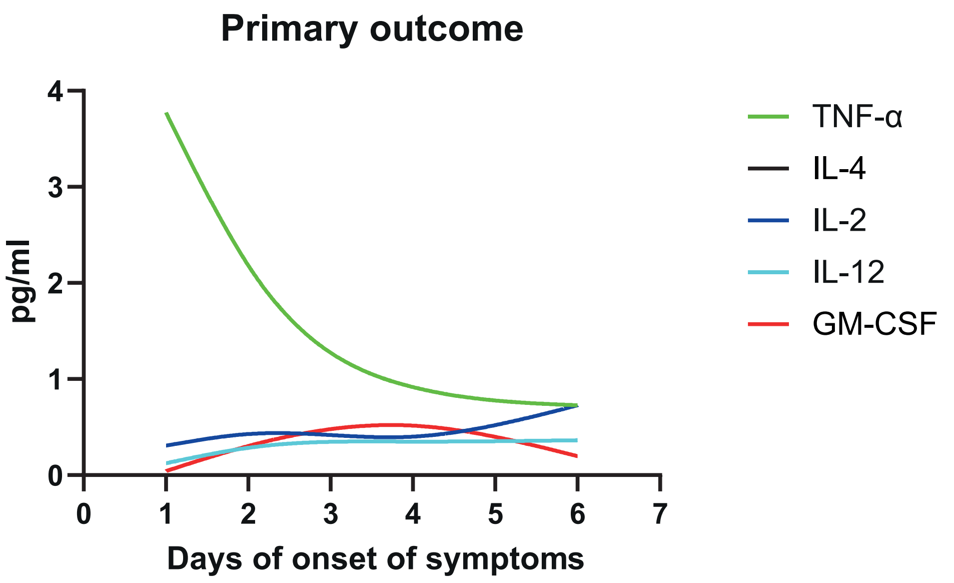

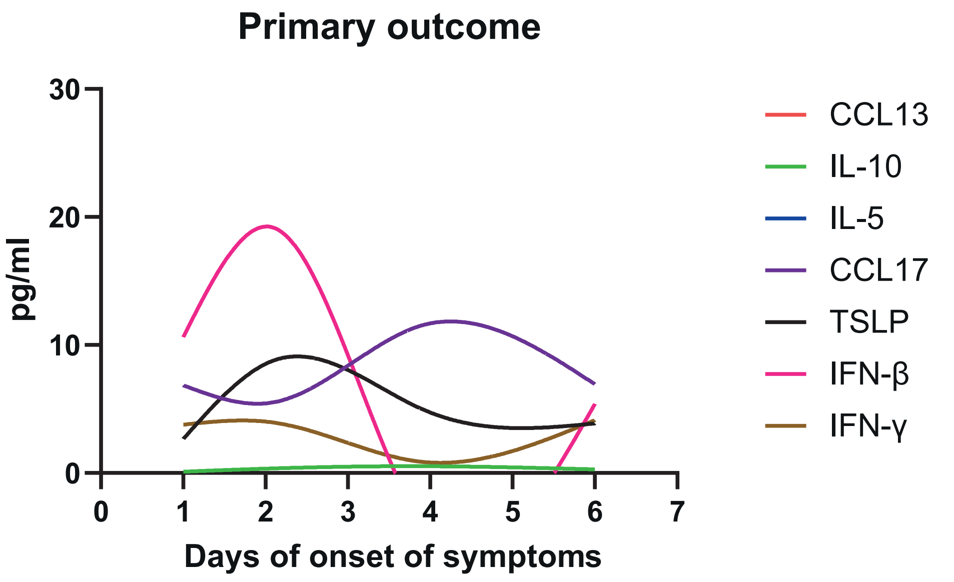

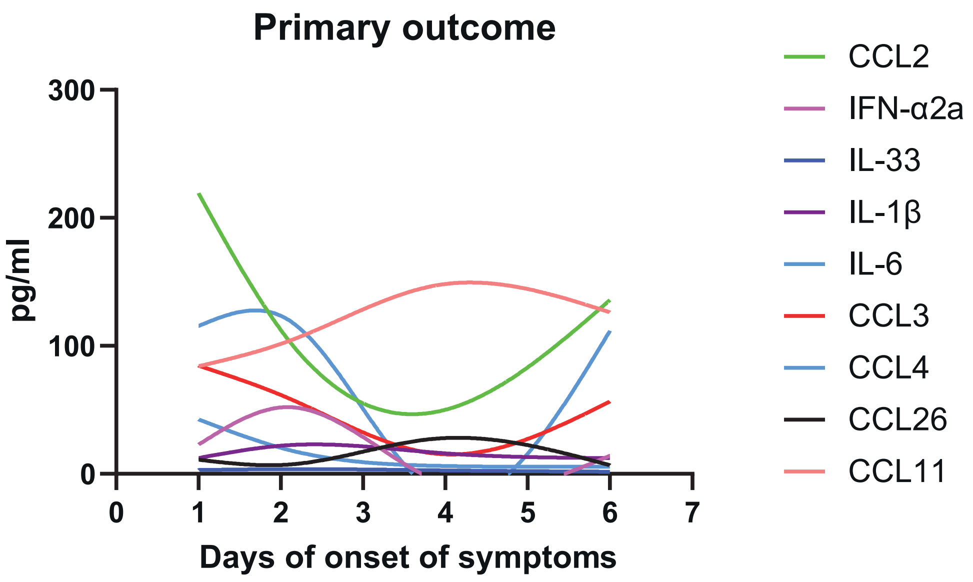
Primary outcome
300
200
100
0
pg/ml
0
1
2
3
4
5
6
7
Days of onset of symptoms
CCL2
IFN-α2a
IL-33
IL-1β
IL-6
CCL3
CCL4
CCL26
CCL11

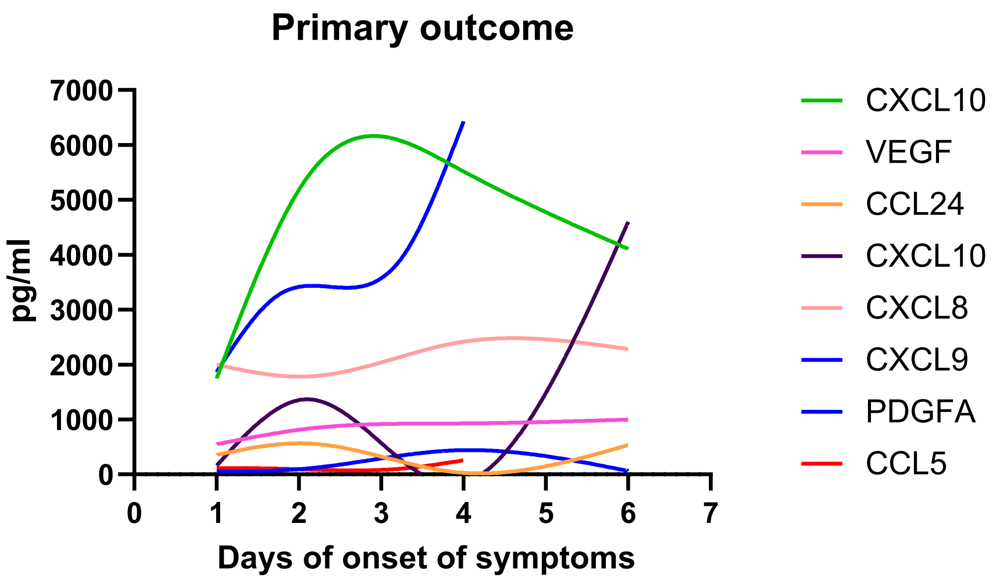
Primary outcome
7000
6000
5000
4000
3000
2000
1000
0
pg/ml
0
1
2
3
4
5
6
7
Days of onset of symptoms
CXCL10
VEGF
CCL24
CXCL10
CXCL8
CXCL9
PDGFA
CCL5

Figure S6. Heatmap for one patient who developed severe COVID-19 infection and met the study primary outcome who gave two nasal samples.

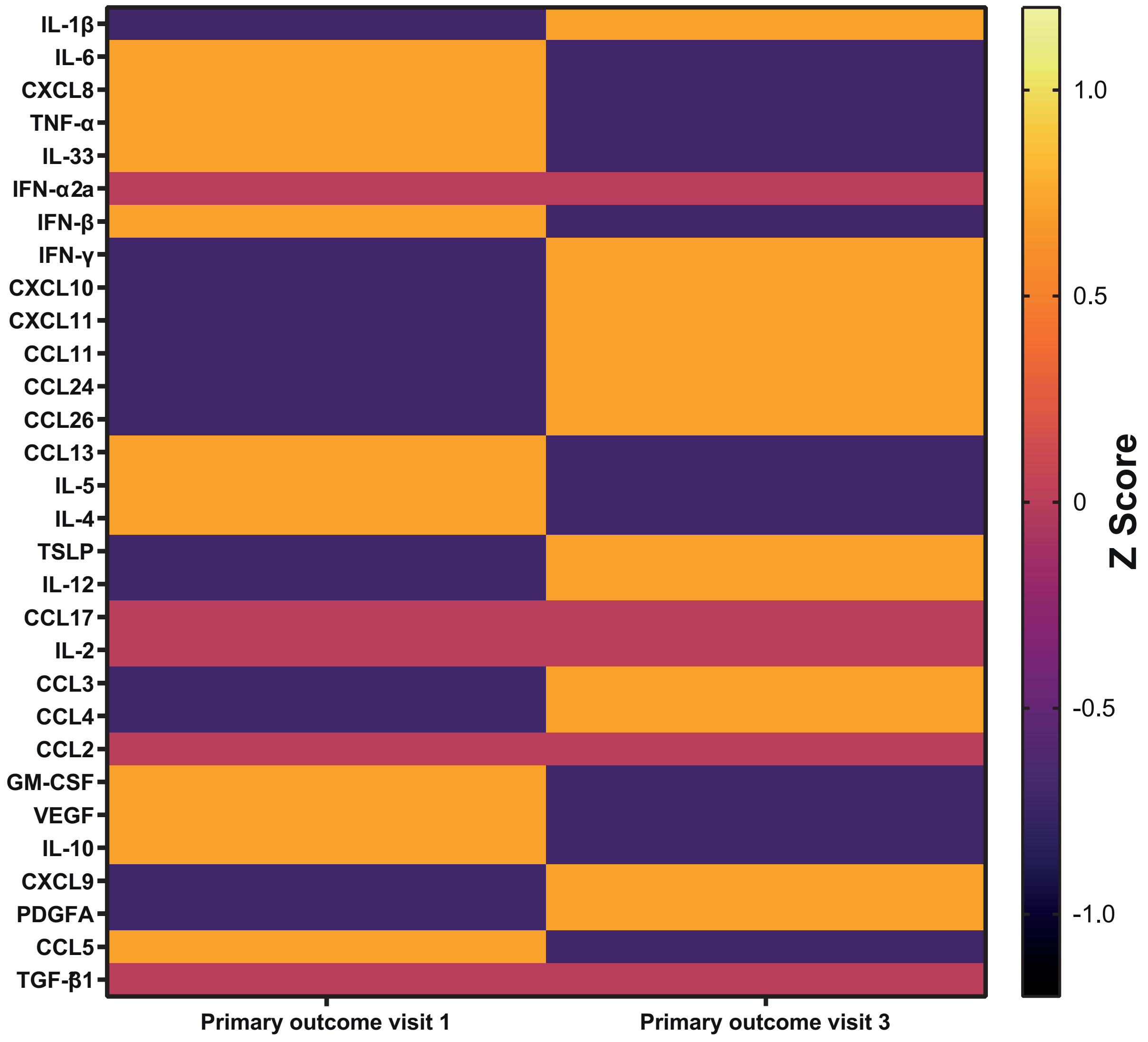

Figure S7. Additional systemic mediators which remain altered 28-35 days after COVID-19 infection. A-K) Violin plots comparing mediator levels in the serum of healthy individuals (n=19), those in the usual care arm (n=61) and budesonide arm (n=62) of the study at 28 days post recruitment. Data were analysed by Kruskal-Wallis with post-hoc Dunn's test. * $p<0.05$, ** $p<0.01$, ***$p<0.001$, ****$p<0.0001$.

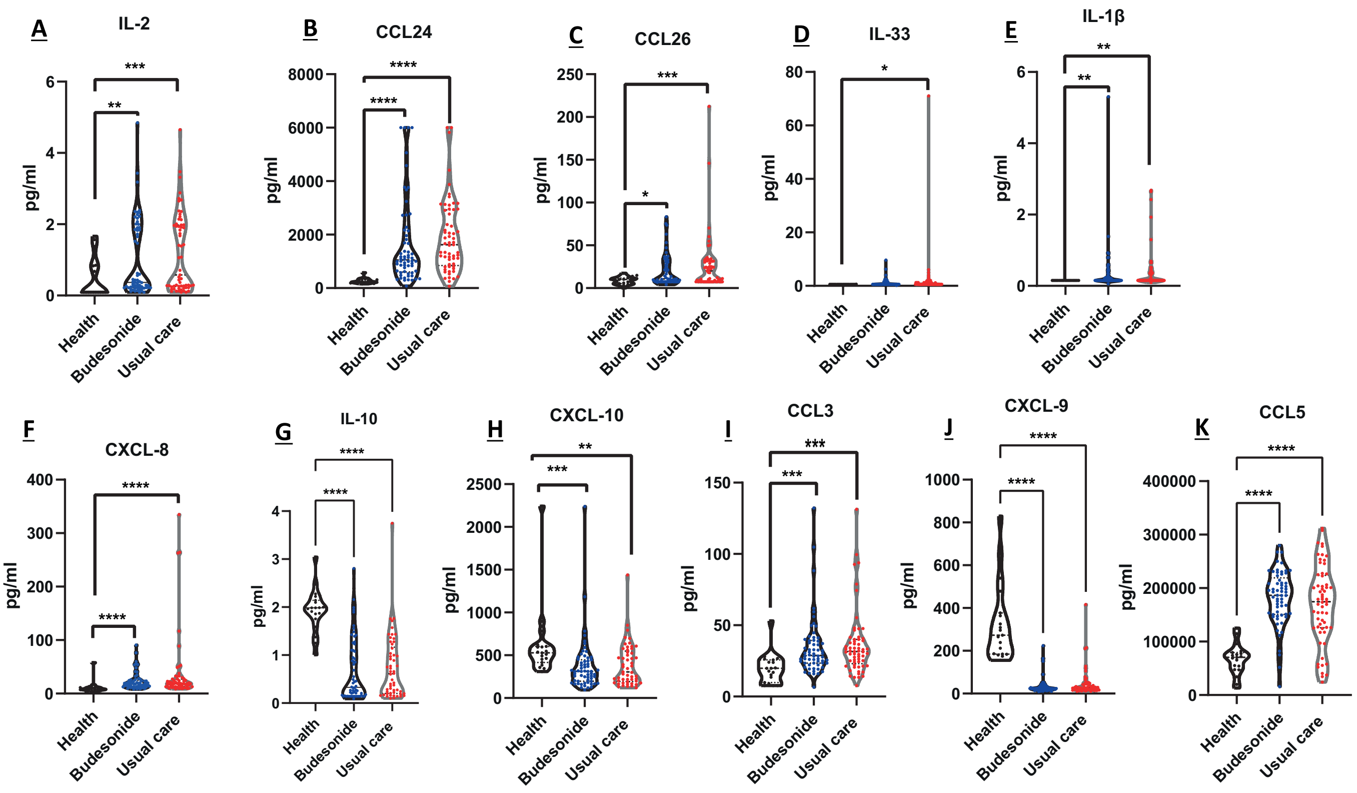

Figure S8. Graphical representation of inflammation and the attenuation of inflammation in early COVID-19.

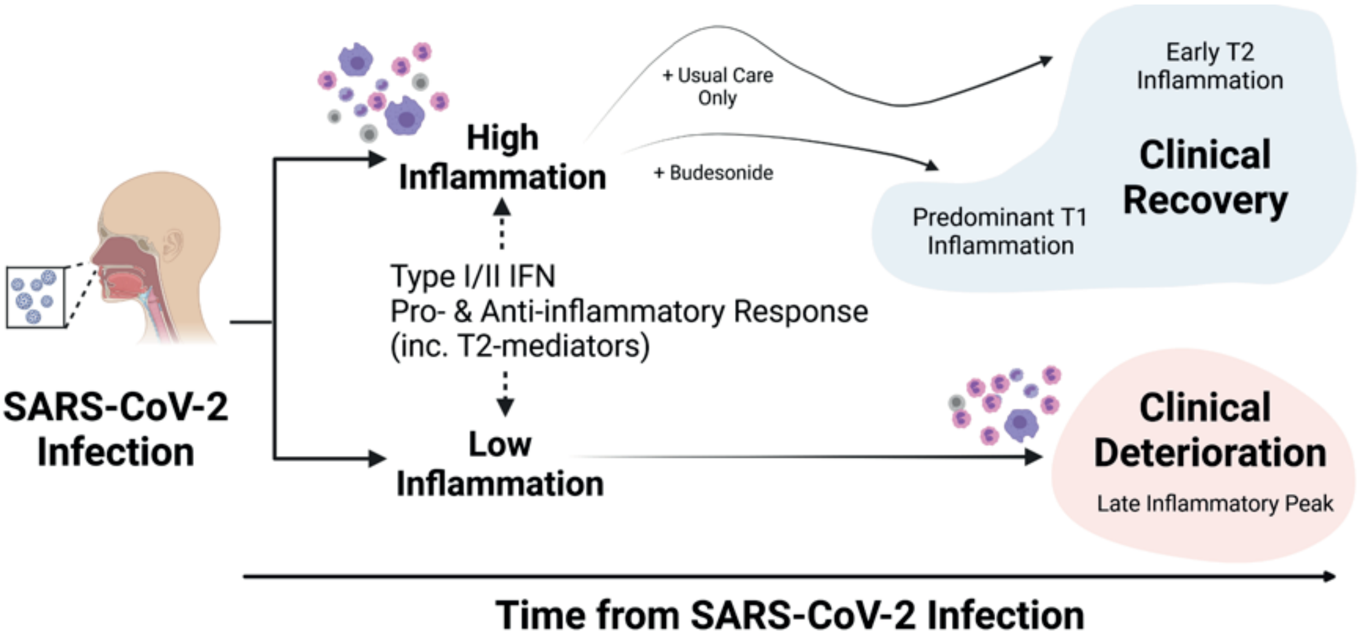

Supplementary Tables

Supplementary Table S1 Demographic table of participants

Characteristic	STOIC COVID-19 participants (n=140)	Healthy controls (n=20)	P-Value
Age, years (SD)	45 (13)	35 (10)	p<0.001
Female sex, no. (%)	84 (60%)	13 (65%)	p=0.500
White ethnicity, no. (%)	131 (93%)	18 (90%)	p=0.500
Median duration of symptoms prior to randomisation, days	3 (IQR 2 - 4)		
Evidence of COVID positive status, no. (%)	134 (95%)		
FLUPRO score at baseline (SD)	0.816 (0.464)		
CCQ score at baseline (SD)	0.707 (0.428)		
Median time to symptom resolution, days (IQR)	9 (6-15 days)		

Supplementary Table S2. Lower and upper limit of detection for measured mediators using MSD immunoassays

Mediator	Lower Limit of Detection (pg/ml)	Upper Limit of Detection (pg/ml)
**IL1B**	0.15	3800
**IL-6**	0.33	2000
**CXCL8**	0.15	2200
**TNF-a**	0.51	3700
**IFN-a2a**	4.00	42400
**IFNb**	3.1	100000
**IFNg**	1.70	17000
**CXCL9**	0.37	80000
**CXCL10**	0.49	6000
**CXCL11**	1.50	5100
**CCL11**	3.20	4800
**CCL24**	3.10	6000
**CCL26**	7.30	21400
**CCL13**	7.50	3800
**IL-2**	0.70	1900
**IL-4**	0.08	2100
**IL-5**	0.24	4000
**IL-10**	0.14	3700
**IL-12**	0.69	2400
**IL33**	0.59	10300
**TSLP**	0.20	10100
**CCL2**	0.74	6600
**CCL3**	7.70	4200
**CCL4**	1.50	1600
**CCL5**	0.25	10000
**CCL17**	0.51	2200
**GM-CSF**	0.12	9400
**VEGF**	2.00	4900
**PDGFA**	2.1	2000
**TGF-B1**	9.1	37000

Supplementary Table S3. Correlation between nasal mediators and viral load. Two tailed Spearman rank correlations with 95% confidence intervals between viral load and individual mediator levels at visit 1 for those that gave both a viral swab and nasosorption sample (n=122). A). Only positively correlated mediated depicted. **= p<0.01, *= p<0.05.

	Viral titre vs. IL-12	Viral titre vs. CXCL10	Viral titre vs. CCL2	Viral titre vs. CXCL11	Viral titre vs. IFN-α2a	Viral titre vs. IL-6
Spearman r	0.2540	0.2443	0.2462	0.2373	0.2242	0.2241
95% confidence interval	0.07454 to 0.4175	0.06428 to 0.4089	0.06626 to 0.4106	0.05688 to 0.4027	0.04308 to 0.3911	0.04300 to 0.3910
P (two-tailed)	0.0048	0.0067	0.0063	0.0085	0.0130	0.0131
P value summary	**	**	**	**	*	*

Supplementary Table S4

pg/ml	Healthy Controls	Early COVID-19	Usual Care arm day 14	Budesonide arm day 14
IL-4	0.08 (0.08 to 0.08)	0.12 (0.10 to 0.14)	0.10 (0.09 to 0.11)	0.10 (0.09 to 0.12)
IL-12p70	0.22 (0.13 to 0.36)	0.54 (0.44 to 0.67)	0.27 (0.20 to 0.36)	0.22 (0.17 to 0.29)
IL-2	0.27 (0.14 to 0.53)	0.83 (0.67 to 1.02)	0.52 (0.37 to 0.73)	0.58 (0.43 to 0.79)
TNF-α	0.56 (0.50 to 0.64)	1.40 (1.18 to 1.66)	1.01 (0.81 to 1.27)	1.06 (0.83 to 1.36)
IL-10	0.59 (0.38 to 0.91)	0.65 (0.49 to 0.86)	0.19 (0.15 to 0.25)	0.18 (0.13 to 0.24)
GM-CSF	0.62 (0.35 to 1.08)	0.37 (0.30 to 0.45)	0.43 (0.32 to 0.58)	0.50 (0.38 to 0.67)
IL-5	2.24 (1.16 to 4.33)	1.54 (1.24 to 1.93)	1.74 (1.17 to 2.58)	2.24 (1.56 to 3.23)
IFN-γ	2.55 (1.74 to 3.73)	5.05 (4.09 to 6.24)	5.25 (3.85 to 7.15)	4.63 (3.47 to 6.16)
IL-6	2.98 (1.65 to 5.40)	9.33 (7.16 to 12.16)	3.76 (2.53 to 5.61)	4.86 (3.23 to 7.33)
IFN-β	3.46 (2.94 to 4.07)	6.88 (5.84 to 8.11)	5.61 (4.58 to 6.86)	5.91 (4.78 to 7.32)
IFN-α2a	4.00 (4.00 to 4.00)	7.49 (6.23 to 9.00)	3.99 (3.89 to 4.09)	4.02 (3.82 to 4.23)
IL-33	7.01 (3.02 to 16.20)	6.93 (4.90 to 9.82)	7.10 (3.92 to 12.86)	5.45 (3.35 to 8.86)
CCL26	9.04 (7.12 to 11.48)	12.50 (10.87 to 14.38)	12.34 (9.81 to 15.53)	12.14 (9.32 to 15.82)
CCL13	10.07 (7.53 to 13.48)	14.86 (12.72 to 17.36)	10.31 (8.60 to 12.35)	12.09 (9.71 to 15.05)
TSLP	14.24 (10.40 to 19.48)	4.39 (3.62 to 5.34)	3.90 (2.78 to 5.48)	4.15 (2.96 to 5.82)
CCL3	15.01 (10.76 to 20.94)	39.41 (32.44 to 47.88)	21.72 (17.34 to 27.21)	23.74 (18.80 to 29.9)
IL-1β	15.33 (9.00 to 26.12)	13.26 (10.43 to 16.87)	15.37 (10.12 to 23.35)	17.12 (11.77 to 24.92)
CXCL11	15.57 (8.33 to 29.11)	240.45 (166.02 to 348.25)	36.86 (26.26 to 51.73)	50.37 (34.73 to 73.06)
CCL4	18.64 (11.93 to 29.13)	53.05 (43.25 to 65.07)	26.93 (21.80 to 33.27)	29.52 (23.26 to 37.46)
CCL17	20.46 (12.90 to 30.12)	6.27 (5.33 to 7.36)	6.58 (5.02 to 8.64)	7.46 (5.50 to 10.11)
CCL5	28.82 (18.29 to 45.39)	1261.45 (937.54 to 1697.28)	875.73 (493.41 to 1554.29)	756.10 (461.05 to 1239.98)
CCL11	34.71 (19.51 to 61.78)	91.59 (77.98 to 107.58)	91.06 (71.46 to 116.03)	107.44 (81.46 to 141.70)
CCL24	119.81 (68.30 to 210.17)	207.23 (172.56 to 248.87)	215.26 (153.66 to 301.56)	259.42 (174.48 to 385.71)
CCL2	129.80 (91.49 to 184.16)	76.77 (63.07 to 93.44)	42.84 (32.97 to 55.66)	46.02 (34.12 to 62.05)
PDGFA	222.88 (118.83 to 418.04)	172.62 (130.03 to 229.15)	127.20 (81.66 to 198.12)	153.37 (99.36 to 236.72)
VEGF	822.24 (608.92 to 1110.30)	537.02 (467.49 to 615.57)	579.71 (451.41 to 744.47)	565.53 (434.13 to 736.69)
CXCL9	856.15 (561.01 to 1306.55)	143.51 (105.81 to 194.63)	68.19 (45.90 to 101.32)	87.92 (58.05 to 133.17)
CXCL10	864.37 (534.98 to 1396.59)	2344.61 (1804.43 to 3046.50)	997.30 (725.02 to 1371.84)	1054.08 (711.89 to 1560.74)
CXCL8	1126.80 (693.622 to 1830.52)	1179.66 (965.95 to 1440.64)	1110.47 (850.16 to 1450.47)	942.61 (668.20 to 1329.73)

Data presented as geometric mean and 95% confidence interval of the geometric mean

References

1. Lucas C, Wong P, Klein J, et al. Longitudinal analyses reveal immunological misfiring in severe COVID-19. *Nature* 2020; **584**(7821): 463-9.
2. Morton B, Barnes KG, Anscombe C, et al. Distinct clinical and immunological profiles of patients with evidence of SARS-CoV-2 infection in sub-Saharan Africa. *Nature communications* 2021; **12**(1): 3554.
3. Thwaites RS, Sanchez Sevilla Uruchurtu A, Siggins MK, et al. Inflammatory profiles across the spectrum of disease reveal a distinct role for GM-CSF in severe COVID-19. *Sci Immunol* 2021; **6**(57).
4. Blondel VD, Guillaume J-L, Lambiotte R, Lefebvre E. Fast unfolding of communities in large networks. *Journal of Statistical Mechanics: Theory and Experiment* 2008; **2008**(10): P10008.
5. Ramakrishnan S, Nicolau DV, Jr., Langford B, et al. Inhaled budesonide in the treatment of early COVID-19 (STOIC): a phase 2, open-label, randomised controlled trial. *Lancet Respir Med* 2021.
